# Regression methods for investigating risk factors of chronic kidney disease outcomes: the state of the art

**DOI:** 10.1186/1471-2369-15-45

**Published:** 2014-03-14

**Authors:** Julie Boucquemont, Georg Heinze, Kitty J Jager, Rainer Oberbauer, Karen Leffondre

**Affiliations:** 1 University of Bordeaux, ISPED, Centre INSERM U897-Epidemiology-Biostatistics, Bordeaux F33000, France; 2 Medical University of Vienna, Center for Medical Statistics, Informatics and Intelligent Systems, Section for Clinical Biometrics, Vienna, Austria; 3 Department of Medical Informatics, ERA-EDTA Registry, Academic Medical Center, Amsterdam, The Netherlands; 4 Medical University of Vienna, Vienna, Austria

**Keywords:** Kidney disease, Progression, ESRD, Survival analysis, Competing risks, Interval censoring, Multistate model, Longitudinal analysis, Mixed models

## Abstract

**Background:**

Chronic kidney disease (CKD) is a progressive and usually irreversible disease. Different types of outcomes are of interest in the course of CKD such as time-to-dialysis, transplantation or decline of the glomerular filtration rate (GFR). Statistical analyses aiming at investigating the association between these outcomes and risk factors raise a number of methodological issues. The objective of this study was to give an overview of these issues and to highlight some statistical methods that can address these topics.

**Methods:**

A literature review of statistical methods published between 2002 and 2012 to investigate risk factors of CKD outcomes was conducted within the Scopus database. The results of the review were used to identify important methodological issues as well as to discuss solutions for each type of CKD outcome.

**Results:**

Three hundred and four papers were selected. Time-to-event outcomes were more often investigated than quantitative outcome variables measuring kidney function over time. The most frequently investigated events in survival analyses were all-cause death, initiation of kidney replacement therapy, and progression to a specific value of GFR. While competing risks were commonly accounted for, interval censoring was rarely acknowledged when appropriate despite existing methods. When the outcome of interest was the quantitative decline of kidney function over time, standard linear models focussing on the slope of GFR over time were almost as often used as linear mixed models which allow various numbers of repeated measurements of kidney function per patient. Informative dropout was accounted for in some of these longitudinal analyses.

**Conclusions:**

This study provides a broad overview of the statistical methods used in the last ten years for investigating risk factors of CKD progression, as well as a discussion of their limitations. Some existing potential alternatives that have been proposed in the context of CKD or in other contexts are also highlighted.

## Background

**Figure 1:**
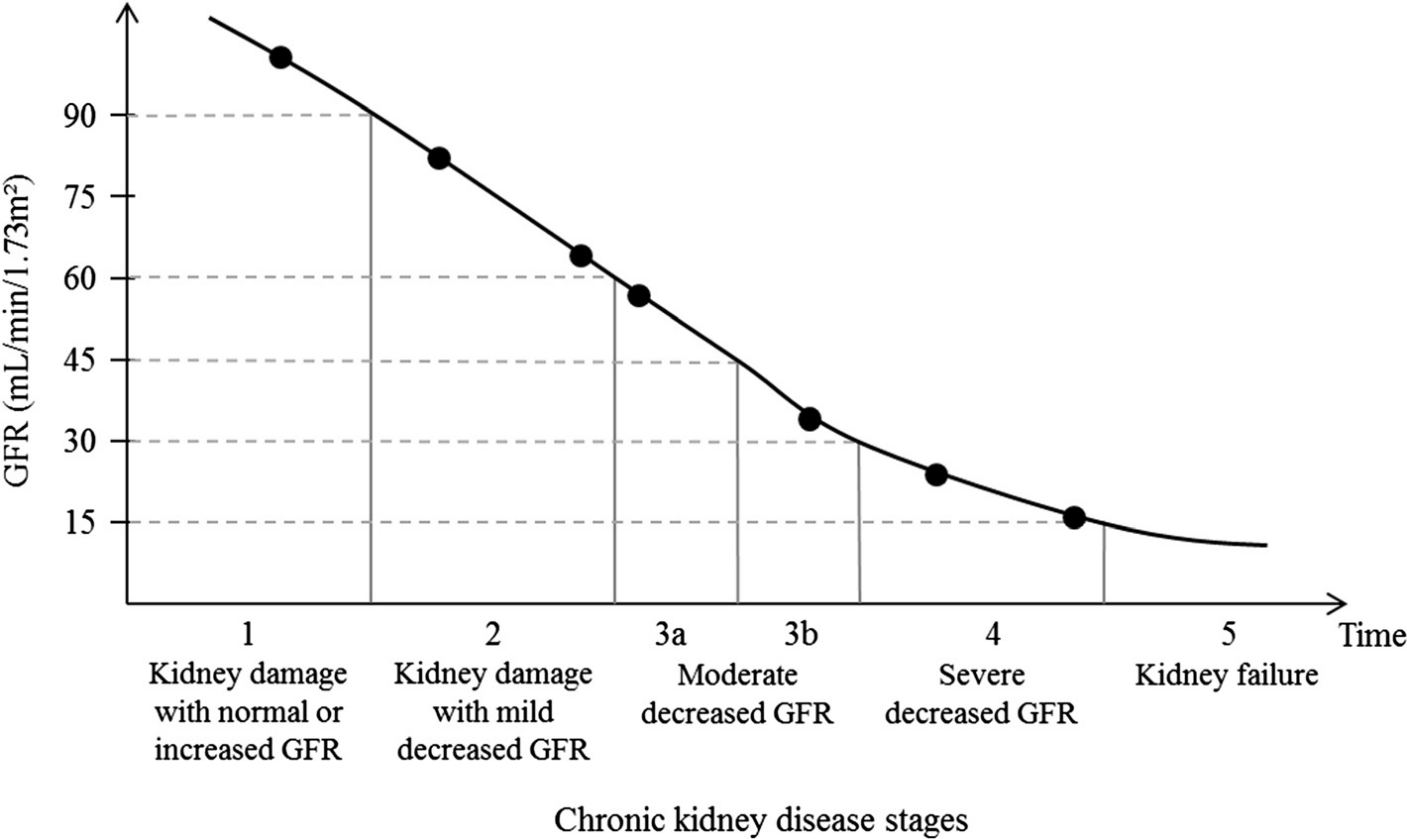
Course of chronic kidney disease for a hypothetical patient with seven measurements of GFR (dots).

Chronic kidney disease (CKD) is a general term for heterogeneous disorders affecting the structure and function of the kidney [1,2]. It usually follows a progressive course and is hardly reversible (Figure 1). There is a need for identification of risk factors of progression of CKD to allow for potential therapeutic interventions. In particular, progression to kidney failure, i.e. a glomerular filtration rate (GFR) of less than 15 mL/min/1.73 m^2^ or the need for treatment with dialysis or transplantation, needs to be prevented because of increased mortality and treatment costs [1].

Various types of outcome variables can be used in the statistical analysis when investigating risk factors associated with CKD progression. For example, the outcome variable can be the time to progression to a specific value of GFR, to initiation of dialysis or transplantation, to cardiovascular events, or to all-cause death. The outcome variable can also be the slope of decline in GFR, or its overall trajectory over time.

If the outcome variable is the time to a particular event of interest, then a survival regression model such as the Cox model may seem obvious to investigate risk factors associated with this event [3]. Standard survival analysis requires the time-to-event to be known exactly for all patients who experience the event. This is the case, e.g. for events such as death or initiation of kidney replacement therapy, where the exact dates can usually be retrieved. However, the time-to-event may not be exactly known for many other events of interest of kidney disease progression. For example, the progression to a specific value of GFR is known only to have occurred between two consecutive measurements of GFR. In such a situation, the time to progression to this value is said to be ‘interval censored’ between the times of these two measurements. Interval censoring should ideally be accounted for in the analysis, especially if the time interval between consecutive measurements is long [4,5]. In addition, survival analysis may have to account for competing risks. A competing event is by definition an event which hinders the observation of the event of interest [6,7]. For example, death is a competing event for any event of progression since patients who die during follow-up can no longer progress after death [8].

If the outcome of interest is the quantitative decline of GFR over time, a linear regression model can be used to investigate the association between risk factors and summary statistics (such as slopes) of the individual evolution of GFR over time. However, such approaches often do not account for all the information available on the repeated measurements of kidney function, and impose some assumptions that may not be valid. A linear mixed model which uses all the repeated quantitative measurements of kidney function may be preferable if there is a sufficient number of patients with at least three measurements [9]. This regression model accounts for correlation between repeated measurements of the same patient, and handles different numbers of measurements per patient that may be measured at unequally spaced intervals, as well as non-linear trajectories over time [10].

Each type of CKD outcome variable thus raises a number of methodological issues in the statistical analysis investigating risk factors. Some of these statistical issues have been acknowledged in the nephrology literature but to our knowledge, no paper gives an overview of these statistical issues. Thus, this paper attempts to provide this overview and to highlight some methods that could address these issues and that have been proposed in the context of CKD or in some other contexts. To this end, we first conducted a literature review of the statistical methods that have been used in the last ten years to investigate risk factors of CKD outcomes. Second, we used the results of this literature review to identify important methodological issues and to highlight some methods that address these issues. The methods used to perform the literature review are described in the next section. In the subsequent results section, we first describe the major CKD outcomes that have been investigated in selected papers, and then describe and discuss the regression methods used for each type of outcomes. Where appropriate, we present some potential alternative analytical approaches that have been never or rarely used in the context of CKD but could yet be of interest.

## Methods

To identify the outcome variables and statistical methods commonly used to investigate the effects of risk factors on progression of CKD, we conducted a literature review restricted to papers written in English language and published from January 2002 to October 1^st^, 2012. We used the Scopus database because it covers a wider range of peer-reviewed journals than most other databases [11]. We performed two searches. Search 1 focused on epidemiological studies (experimental or not) of CKD progression, while Search 2 focused on developments of new statistical methods with application in the field of nephrology. We searched for the same terms in Searches 1 and 2 but we specified different positions of these terms in the papers. Specifically, one of the selected CKD terms (see Table 1) had to appear in the title for Search 1; and in the title, key words, or abstract for Search 2. Selected terms on statistical methods (Table 1) had to appear in either the title, keywords, or abstract for Search 1, while they had to appear in the title for Search 2. To decrease the number of papers in Search 1 and to focus on papers investigating risk factors of CKD outcomes, epidemiological terms on the study design were also required to appear in addition to the keywords of Search 1 (Table 1). To avoid duplication of papers, Search 2 was restricted to papers not identified in Search 1.

**Table 1:** Terms used in our review to identify statistical methods used to investigate risk factors of CKD outcomes

**General topic**	**Specific terms used**	**Position in the paper**
**CKD**	• Chronic kidney disease, CKD	Title in Search 1
	• Kidney function, renal function	
	• Glomerular filtration rate, GFR	Title, key words or abstract in Search 2
	• Albuminuria, proteinuria	
	• Kidney disease, renal disease	
	• Dialysis, end-stage renal disease, ESRD, kidney transplant	
**Statistical method**	• Proportional hazard(s), Cox, time-to-event analysis(es), accelerated failure time	Title, key words or abstract in Search 1
	• Frailty, shared	Title in Search 2
	• Competing	
	• Joint	
	• Linear regression(s), linear model(s)	
	• Logistic regression(s), logistic model(s)	
	• Generalized, GEE	
	• Mixed model(s), mixed effect(s)	
	• Poisson	
	• Multi(−)state(s), illness-death, Markov	
	• Trajectory(ies)	
	• Latent	
	• Longitudinal	
	• Mixture, GMM	
**Study design**	• Case–control, cohort, clinical trial, prospective, retrospective	Key words in Search 1
		Not specified in Search 2

*Abbreviations:CKD* chronic kidney disease, *GFR* glomerular filtration rate, *ESRD* end-stage renal disease, *GEE* generalized estimating equations, *GMM* growth mixture model.

We screened the titles and abstracts of all articles resulting from Searches 1 and 2 to include papers only if they investigated risk factors for progression of CKD. Papers were excluded if they investigated only (i) non CKD outcomes in patients with kidney disease or outcomes occurring after kidney replacement therapy only, (ii) non CKD patients (such as patients with acute kidney injury), (iii) kidney disease or renal function as a risk factor for another disease, (iv) performance of equations used to estimate GFR, or (v) risk factors for incidence or prevalence of kidney disease without notion of progression thereafter, or (vi) for other specific reasons (case analysis, economy, chemistry, study/program design, sociology, fundamental sciences, risk prediction, description of population).

For each of the selected articles, we retrieved the outcome variables and the statistical method used to investigate risk factors associated with these outcomes.

## Results and discussion

**Figure 2:**
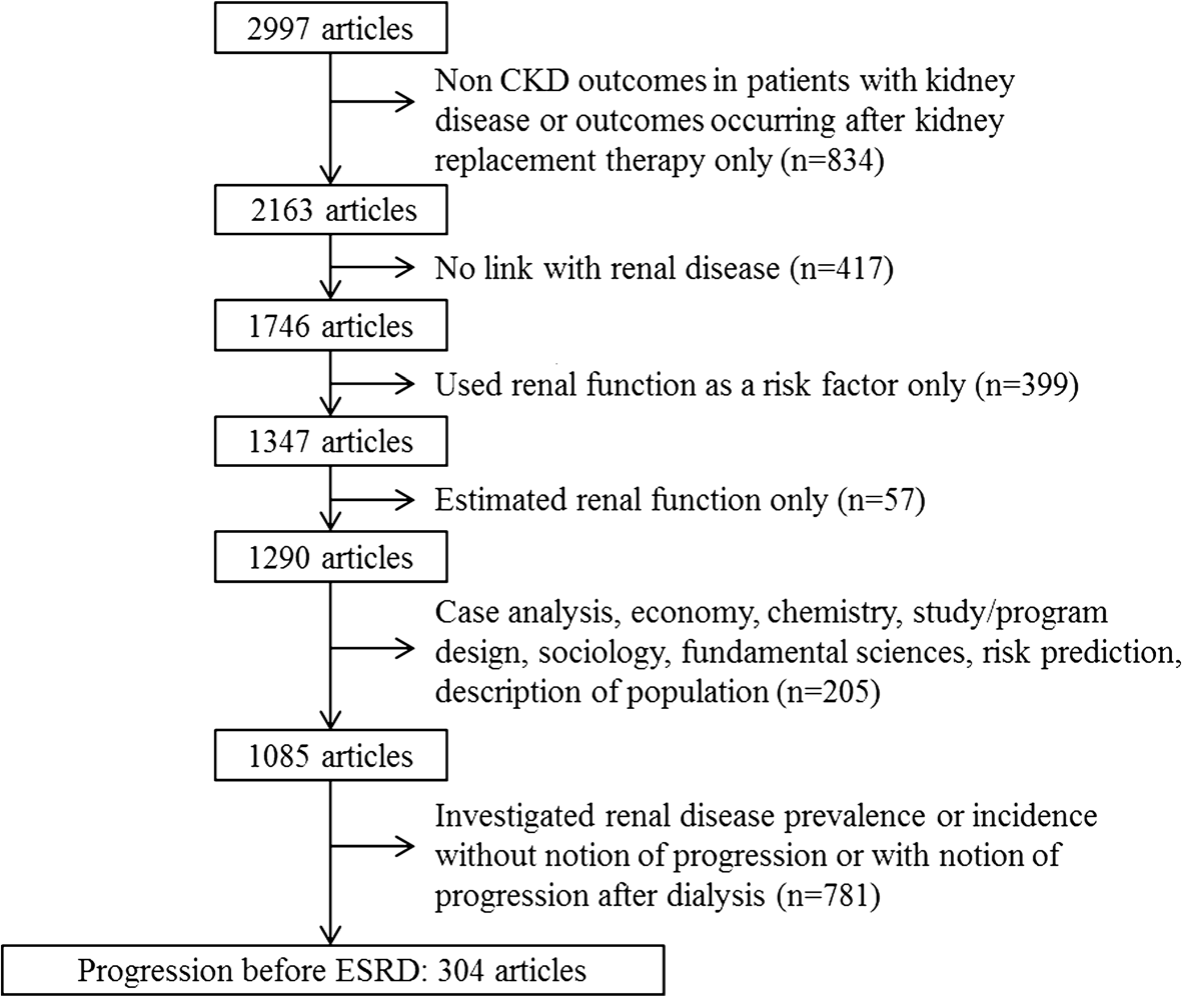
Flow diagram of selected articles.

Search 1 resulted in 2384 papers, and Search 2 identified 613 additional papers. The title and abstract of all 2997 papers were further screened for inclusion and exclusion criteria mentioned in the method section. This resulted in 304 articles that were finally selected for review (Figure 2). See the Additional file 1 for the complete reference list of the 304 selected papers.

Most papers were published in nephrology journals (69%): Nephrology Dialysis Transplantation (20%), the American Journal of Kidney Disease (18%), the Clinical Journal of the American Society of Nephrology (13%), Kidney International (12%) and the Journal of the American Society of Nephrology (11%). Other papers were published in general medical journals (8.5%), journals devoted to cardiology (5.6%), diabetes (3.3%), statistical methods (3%), HIV-AIDS (1.3%), and transplantation (0.3%). The few remaining were selected from journals devoted to other specific subjects (e.g. gerontology, nutrition, pharmaco-epidemiology).

### Major outcomes used in the selected papers

The major outcomes investigated in the 304 selected papers are described in first column of Table 2 for time-to-event outcomes and of Table 3 for quantitative repeated measurements of kidney function. The second column of each table provides the occurrence of the specific outcome among the 304 papers. The sum of occurrences exceeds 304 because many papers investigated several outcomes.

**Table 2:** Frequency of survival (Cox, cause-specific, or Fine and Gray) and logistic regression models used to investigate risk factors of time-to-event outcomes

**Outcome investigated**	**n**^**a**^	**Regression model mentioned in the paper**	**n (%)**^**b**^
**Exactly known time-to-event**	307		
All-cause death	132	Cox model	108 (81.8)
		Cause-specific model	10 (7.6)
		Fine and Gray model	10 (7.6)
		Logistic model	4 (3.0)
Cardiovascular death	31	Cox model	29 (93.6)
		Cause-specific model	1 (3.2)
		Fine and Gray model	1 (3.2)
Cardiovascular event	23	Cox model	23 (100.0)
Initiation of kidney replacement therapy or death due to kidney failure	83	Cox model	65 (78.3)
		Fine and Gray model	10 (12.1)
		Cause-specific model	7 (8.4)
		Logistic model	1 (1.2)
Initiation of kidney replacement therapy or death (whichever comes first)	38	Cox model	38 (100.0)
**Interval-censored time-to-event**	45		
Absolute or relative change in renal function higher than a specific value as compared to baseline value, based on	23	Cox model	7 (30.4)
		Fine and Gray model	1 (4.4)
- GFR (n = 19)		Logistic model	15 (65.2)
- creatinine clearance (n = 3)			
- proteinuria (n = 1)			
Transition to a specific stage of disease, based on	13	Cox model	9 (69.2)
- GFR (n = 9)		Logistic model	4 (30.8)
- proteinuria (n = 4)			
Doubling of creatinine (serum or clearance)	8	Cox model	7 (87.5)
		Logistic model	1 (12.5)
Composite of	1	Cox model	1 (100.0)
**-** decline in 30% of creatinine clearance			
- increase in proteinuria > 3.5 g/d			
**Composite of exact and interval-censored time-to- events**	43	Cox model	35 (81.4)
		Fine and Gray model	3 (7.0)
		Cause-specific model	3 (7.0)
		Logistic model	2 (4.6)

*Abbreviations*: *GFR* glomerular filtration rate, *CKD* chronic kidney disease.

^a^Number of occurrences the specific outcome was used. The total exceeds 304 because some papers investigated several types of events.

^b^Number and percentage of occurrences the statistical method was used for each specific outcome.

**Table 3:** Frequency of standard linear, linear mixed, and generalized estimating equations regression models to investigate repeated measurements of renal function

**Outcome investigated**	**n**^**a**^	**Regression model mentioned in the paper**	**n (%)**^**b**^
**All repeated measurements of renal function**	48		
Repeated measurements of	36	Linear mixed model	22 (61.1)
- GFR (n = 33)		Linear mixed model accounting for informative	8 (22.2)
- Creatinine clearance (n = 2)		drop-out	
- Proteinuria (n = 1)		Linear GEE	4 (11.1)
		Linear GEE accounting for informative drop-out	1 (2.8)
		Latent class growth analysis	1 (2.8)
Repeated measurements of	10	Linear mixed model	7 (70.0)
- log GFR (n = 5)		Linear GEE	2 (20.0)
- log creatinine (serum or clearance) (n = 2)		Latent class growth analysis	1 (10.0)
- log proteinuria (n = 3)			
Absolute GFR change between each visit and baseline	1	Linear mixed model	1 (100.0)
Relative GFR change each year	1	Linear GEE	1 (100.0)
**A summary statistic for the change of renal function**	45		
Individual slope^c^ of	36	Linear model	36 (100.0)
- GFR (n = 30)			
- Creatinine (serum or clearance) (n = 4)			
- UACR (n = 2)			
Absolute GFR change as compared to baseline	7	Linear model	7 (100.0)
Relative GFR change as compared to baseline	1	Linear model	1 (100.0)
Log of absolute proteinuria change as compared to baseline	1	Linear model	1 (100.0)

*Abbreviations:GFR* glomerular filtration rate, *GEE* generalized estimating equations, *UACR* urine albumin-to-creatinine ratio.

^a^Number of occurrences the specific outcome was used. The total exceeds 304 because some papers investigated several types of outcomes.

^b^Number and percentage of occurrences the statistical method was used for each specific outcome.

^c^Slope of a marker is a summary statistic derived from measurements of a patient.

Time-to-event outcomes were separated into three subgroups. A first subgroup was comprised of outcomes for which the time-to-event was known exactly (n = 307 occurrences, Table 2). A second subgroup was comprised of outcomes for which the time-to-event was interval censored between two consecutive measurements of kidney function (n = 45). A third subgroup comprised time-to-event outcomes combining at least one event for which time-to-event was exactly known and one event for which time-to-event was interval censored (n = 43). Many papers mentioned incidence of end-stage renal disease (ESRD) as the event of interest. However, the definition of ESRD varied across the papers. While in some papers incidence of ESRD was defined as the initiation of kidney replacement therapy (either dialysis or transplantation, whichever came first), others defined it as the initiation of dialysis only, transplantation only, death due to kidney failure only, or any of these events whichever came first. Overall, all of the events for which dates were known exactly were investigated on 83 occasions (Table 2). Initiation of kidney replacement therapy or all-cause death, whichever came first, was investigated on 38 occasions (Table 2). When ESRD was defined as a GFR < 15 ml/min/1.73 m^2^, time-to-event was interval-censored between two consecutive measurements of GFR, and was then classified into the second or third subgroup of events, depending on whether it was combined or not with another event for which the date was known exactly such as initiation of kidney replacement therapy (Table 2). The most frequent event in the second subgroup was an absolute or a relative decrease in GFR superior to a specific value, compared with the baseline GFR value (n = 19, Table 2).

In the 304 selected papers, the quantitative measurement of kidney function over time was the outcome of interest on 93 occasions (Table 3). The most frequently investigated measurement was GFR (n = 78 occurrences) but authors also investigated creatinine (n = 8) or proteinuria (n = 7). While all individual repeated measurements of the kidney function over time were used on 48 occasions, a summary statistic such as the individual slope of the marker over time was used as the outcome variable on 45 occasions (Table 3).

### Regression methods used in the selected papers and related statistical issues

The regression methods used for each outcome are reported in the third column of Tables 2 and 3. The last column indicates the occurrence and percentage of the use of the regression method among the papers investigating the specific outcome. The papers that used each of the specific regression models are listed in Table 2 available in the Additional file 1. Below, we describe these methods and discuss potential issues and alternative methods that have been proposed in the context of CKD or in other contexts. Furthermore, Table 4 provides a list of some statistical procedures that are available in SAS, R, or STATA software to perform the most advanced statistical analyses discussed below.

**Table 4:** Examples of available software that handle statistical challenges in progression of CKD

**Statistical issue**	**Software**		
	**SAS**	**R**	**STATA**
**Exactly known time-to-event outcome**			
Survival regression models	PROC PHREG	survival	stcox
Competing risks models			
* Cause-specific model*	PROC PHREG	survival	stcox
* Fine and Gray model*	PSHREG macro	cmprsk	stcrreg
Multistate models	PROC PHREG	mstate	stcox
		msm	
		tdc.msm	
**Interval-censored time-to-event outcome**			
Survival regression models	PROC LIFEREG	intcox	intcens
	EMICM macro	survival	
	ICSTEST macro	SmoothHazard	
	ICE macro		
Competing risk with death		SmoothHazard	
		msm	
Multistate models		msm	
**Quantitative outcomes**			
Generalized estimating equations	PROC GENMOD	gee	xtgee
		geepack	
		yags	
Mixed models	PROC GLIMMIX	lme	xtmixed
	PROC MIXED	glmer	GLLAMM
	PROC NLMIXED		
Identification of subpopulation of trajectories			
*Latent class growth analysis*	PROC TRAJ		
*Latent class mixed model*	SASRTM macro	lcmm	GLLAMM
Informative drop-out censoring			
*Shared random-effects models*	PROC NLMIXED	jm	jmre1
	CGEE2 macro		
* Joint latent class models*		lcmm	

#### Time-to-event outcomes

Only few analyses used logistic regression for time-to-event outcomes, except when investigators considered a specific absolute or relative change in renal function over a given period of time as an event of interest (n = 15 occurrences, 65.2%, Table 2). Logistic regression analysis assumes no drop-out due to loss-to-follow-up or death during that period of time. The most popular method to account for individual follow-up times was the standard survival Cox model, as expected. For example, on the 132 occurrences where all-cause death was investigated, the Cox model was mentioned 108 (81.8%) times. Among the 83 occurrences where initiation of kidney replacement therapy or death due to kidney failure was investigated, the Cox model was mentioned 65 (78.3%) times.

##### Accounting for competing risks

The cause-specific proportional hazards model and the Fine and Gray model (also called proportional subdistribution hazards model) are two regression methods often used to account for competing risks [8,12]. For example, among the 132 studies investigating risk factors of all-cause death, these competing risks models were used 20 times (15.2%) to account for initiation of kidney replacement therapy (see e.g. [8,13,14]). If one wants to investigate risk factors of all-cause death, kidney replacement therapy can indeed be considered a competing event if one is interested in all-cause-death before kidney replacement therapy. The cause-specific proportional hazards model and the Fine and Gray model were also used to account for death in studies exploring factors associated with initiation of kidney replacement therapy (n = 17, 20.5%) because patients who die before initiation of therapy can no longer initiate therapy after death.

In the cause-specific proportional hazards model, the time-to-event of interest for patients who experience the competing event is censored at the time of the competing event occurred, if the latter occurs before the event of interest. Any software that handles the Cox model can be used to estimate a cause-specific proportional hazards model, as indicated in Table 4. The only requirement is to provide correct time-to-censoring for patients who experience the competing event before the event of interest. Many of the papers which mentioned the use of the Cox model actually used a cause-specific proportional hazards model. For example, on the 108 occurrences where authors mentioned the use of the Cox model to investigate risk factors of all-cause death, censoring at initiation of kidney replacement therapy was reported on 26 occasions (24.1%). On the 65 occurrences where authors mentioned the use of the Cox model to investigate factors associated with initiation of kidney replacement therapy, censoring at death was reported on 32 occasions (49.2%). Similar results were observed for other time-to-event outcomes. Competing risks were thus taken into account more frequently than what Table 2 may suggest at first glance. The major advantage of the cause-specific proportional hazards model is that regression coefficients have a hazard (i.e. rate) ratio interpretation [6,12,15], which is useful to study the aetiology of diseases.

In contrast to the cause-specific proportional hazards model, in the Fine and Gray model, patients who experience the competing event are artificially retain in the risk sets even after the competing event occurred, with decreasing weights over time. Therefore, the regression coefficients do not have a rate ratio interpretation. However, they are useful for prediction since they have a direct relationship with the cumulative incidence functions which measure the risk (*i.e.* probability) of an event within a given time interval [6,12,15]. The Fine and Gray model is implemented in several statistical packages (see e.g. the SAS PHSREG macro [16]) as indicated in Table 4.

Some alternatives to the cause-specific proportional hazards model and to the Fine and Gray model have been proposed and used in the context of CKD. For example, Cianciaruso *et al.*[17] used a marginal competing risks model and Scolari *et al.*[18] used a frailty model to account for the correlation between ESRD and death times [19]. However, it should be mentioned that independence of competing risks is not needed for a valid inference of the cause-specific model [15].

All the competing risk analyses mentioned above require both the time-to-event of interest and the time-to-competing event to be known exactly. While this usually holds for the time to initiation of renal replacement therapy and the time to death, this is never the case for the time to a specific stage of CKD based on GFR measurement as discussed in the two following subsections.

##### Accounting for interval censoring

As mentioned in the introduction, interval censoring occurs in survival analysis when the event of interest is lacking a precise date and is only known to have occurred between two dates [4,20]. For example, if the event of interest is transition to stage 3a of CKD, *i.e.* a GFR falling below 60 mL/min/1.73 m^2^ (see Figure 1), the time to event is interval censored between the last measurement with a GFR above 60 mL/min/1.73 m^2^ and the first measurement with a GFR below 60 mL/min/1.73 m^2^. For example, for our hypothetical patient, we only know that progression to stage 3a occurred between his or her third and fourth GFR measurement. Of the 24 occurrences where the Cox model was used to investigate risk factors of a single interval-censored time-to-event (Table 2), the interval censoring issue was never acknowledged. Authors actually imputed the time-to-event by the time to the first measurement where the marker was below the specific level of interest (e.g. 60 mL/min/1.73 m^2^ for GFR), or did not specify how they imputed it. Yet such imputation may produce biased estimates of risk factor effects and standard errors if the time intervals between consecutive measurements are long, as they can be for some patients (see e.g. [21-23]). The interval censoring issue has already been acknowledged in the context of CKD, as in Bilous *et al.*, for example, who studied incidence of micro-albuminuria [20]. Several estimation methods handling interval censoring are now available in different statistical software (see Table 4), which should encourage investigators to account for this issue in their analyses. However, interval censoring further complicates competing risks analyses as discussed in the next subsection.

##### Accounting both for competing risks and interval censoring - multistate approach

If one wants to investigate risk factors of progression to a specific stage of CKD based on GFR measurement, e.g. progression to stage 3a, one may have to account for both interval censoring of the time-to-progression and competition with death. In the cause-specific hazards model, the time to stage 3a should be censored at the time of death for patients who die before being diagnosed with a GFR below 60 mL/min/1.73 m^2^. However, the GFR level at death is usually unknown. Only the last GFR measurement before death is known to be higher than 60 mL/min/1.73 m^2^. Thus, it is uncertain whether the patient had or had not progressed to stage 3a between the last measurement and death. While censoring at death is likely to produce bias because it assumes that the patient did not progress to stage 3a between the last GFR measurement and death, censoring at the last measurement before death might not be a better solution. Indeed, a simulation study has recently shown that censoring either at death or at the last visit produces biased estimates of the effect of factors that are associated with the competing event (death) [24].

An alternative regression method that accounts for the probability to progress to the specific CKD stage of interest between the last measurement of renal function and death or latest follow-up on vital status, is the illness-death model for interval-censored data [5,24]. The illness-death model is a specific multistate model with three states [25]. Consider a hypothetical study where patients would be in CKD stages 1 or 2 at baseline, and the outcome of interest would be transition to stage 3a with death as a competing event. The three states in the illness-death model would be stages 1–2 of CKD (State 0), stage 3a of CKD (State 1), and death (State 2). Using an illness-death model for interval-censored data as the one implemented in the SmoothHazard R package [26] (Table 4) would allow us to account for the fact that patients in the initial state 0 at the last measurement before death may have progressed through the intermediate state 1 of interest before dying. Such a flexibility has been shown to produce accurate estimations of the effects of factors on the state of interest [24]. However, note that this model requires follow-up information on vital status after transition to the specific stage of interest.

When more than one stage is of interest (for example, if one wants to study all stages of CKD) and the follow-up is long enough to ensure a sufficient number of patients observed at different stages of the disease, even more complex multistate models can be used. For example, Begun *et al.* recently considered a six-state model for interval-censored data, where the states were CKD stages 3, 4 and 5, dialysis, transplant, and death [27]. Foucher *et al.* proposed four-state and five-state models for the study of kidney transplant evolution [4,28], considering interval-censored data since intermediate states were defined on creatinine clearance and/or proteinuria levels. Hu *et al.* also proposed a multistate approach to estimate the probabilities to be in CKD stages 1–2, 3 and 4, dialysis, or death before dialysis at different time points, while accounting for the fact that estimated GFR was measured at some specific time points only [29]. Multistate models are now implemented in several statistical software such as the R package mstate [30] (Table 4). However, when the time to progression to some states is interval censored, which is typically the case for the time to all intermediate CKD stages, the estimation procedure is much more complex. Indeed, the states are observed only at some points in time and thus all possible transitions between these time points need to be considered. To our knowledge, only the msm R package [31] handles general multistate models for interval-censored data (Table 4).

#### Quantitative outcome variables measuring kidney function over time

##### Using a single summary statistic as the outcome variable

Among the 45 occurrences where the outcome variable was a summary statistic of the renal function decline over time, individual slope of GFR was used on 36 occasions, and individual change in GFR compared to baseline was used on 7 occasions. All these analyses consist of two steps. In step 1 the summary statistic for each patient is derived, and step 2 uses this summary statistic as the outcome variable in a linear regression to investigate the association with patients’ characteristics at baseline (Table 3). As mentioned by Rosansky [32], using the slope of renal function for measuring renal trajectory may be considered as “a starting point in application of renal trajectory to clinical management”. However, the two-step approach mentioned above is not the best statistical approach to achieve this objective. Indeed, it has numerous disadvantages including an important loss of information [33], especially if individual values of the summary statistic (e.g. slope) are not first estimated using all available measurements per patient or if many patients have only a single measurement of the renal function. Another important issue for statistical inference is that Step 2 does not account for uncertainty of the summary statistic derived in Step 1. Yet, the summary statistic (e.g. slope) is an estimate, and the accuracy of this estimate for a given patient strongly depends on the number of measurements available for that patient, as well as the time points at which these measurements were taken [34]. Ignoring such uncertainty is likely to produce unreliable confidence intervals of the effect of the risk factor on the slope. Finally, using the slope makes the implicit assumption of a linear trajectory of the quantitative marker for each individual, although this may not apply to a large number of CKD patients [35]. Among the 36 occurrences where the slope was used as the summary statistic, 16 (44.4%) had three or more repeated measurements of renal function per patient, which would have made it possible to use more appropriate statistical methods as described in the next section.

##### Using all information and accounting for correlation within individual repeated measurements of the renal function – linear mixed models and GEE

Two types of regression models directly handle outcome variables that have several observed values per subject: mixed models and population average models estimated by generalized estimating equations (GEE; also known as the marginal approach). The effects of the factors on the mean trajectory are estimated in one step only, without the need to first derive a summary statistic for each patient. Of the 48 occurrences where all repeated quantitative measurements of renal function were used as the outcome variable, authors mainly used linear mixed models (n = 38, 79.2%) (see e.g. [9,17,36,37]) to investigate risk factors associated with the outcome. The term mixed models refers to the use of both fixed and random effects in the model. Random effects are used in mixed models to represent variability between patients’ trajectories and to account for correlation between measurements of a same patient. Indeed, this correlation between repeated measurements has to be taken into account for valid inference [10]. The major advantage of linear mixed models is that they require neither equally spaced time intervals between consecutive measurements, nor the same number of measurements per patient. As a result, all available information is used in the estimation process, including patients who have only one available measurement of the outcome. This optimal use of information allows more accurate estimates of the effects of risk factors on the trajectory. Linear mixed models can also handle nonlinear mean trajectories over time of the marker.

On eight occasions (21.1%), authors used GEE (see e.g. [38]). As all regression models, GEE handles any kind of explanatory variables (see [39] for a general discussion of representation of explanatory variables in regression models). As mixed models, GEE account for correlations between repeated measurements of a same patient [33]. When used to model individual repeated values of a quantitative marker of the renal function, both linear mixed models and linear GEE can be used to investigate risk factors associated with the mean trajectory of the marker over time. The estimation method differs, but in the context of linear models, they should yield to very similar estimates of risk factor effects, provided the set of covariates as well as the correlation structure between repeated measurements are equivalent in both approaches. However, as opposed to standard mixed models, standard GEE assumes that any study dropouts are completely random. This assumption does not hold if some patients are early dropouts because of dialysis or transplantation. Indeed, such a dropout is not completely random since it is highly related to the rate of decline in GFR [40] (see section on informative dropout). In addition, because mixed models allow the modelling of individual trajectories over time (via random effects), they are more appropriate to perform individual prediction than GEE. Indeed, GEE do not model individual departure from the average trajectory over time. Mixed models may thus be more interesting in a clinical setting. Both methods are implemented in most standard statistical software (Table 4). However, mixed models may appear to be more complicated to use than GEE because of the need to understand the concept of random effects, and the need to specify their distribution. Misspecification of the random effect distribution may in some situations bias the results, but some solutions have been proposed and applied to CKD data [41].

##### Accounting for non-normality of the measurements of the renal function

When using standard techniques to estimate linear models, linear mixed models or linear GEE, one of the four fundamental assumptions is the normal distribution of errors. This assumption of normality must be checked on the distribution of the residuals from the estimated model [42]. If this assumption does not hold, a suitable transformation of the outcome may be needed. Of the 91 occasions where the outcome of interest was a quantitative measurement of the renal function, a log-transformation of the measurement (GFR, creatinine, proteinuria, or proteinuria change) was used on 11 occasions for this reason. However, the regression coefficients have a different interpretation after such a transformation of the outcome variable since they measure the impact of risk factors on the log of the renal function, and not directly on the renal function. Furthermore, the results of the linear model or the linear mixed model may be robust to some departure from normal distribution, especially for large sample sizes [43]. In some situations, one may therefore have interest in not transforming the measurement of kidney function in order to facilitate interpretation and comparison of results between studies.

##### Investigating subpopulations of trajectories of the quantitative marker

The linear mixed model or the population average model estimated by GEE assumes a homogeneous population, i.e. only one mean trajectory within the population. In CKD, several studies have shown that the assumption of only one homogeneous population in terms of renal function trajectory over time could be too strong. For example, Li et *al.* have recently shown that not all patients with CKD have a steady GFR progression over time [35]. Different statistical methods can be used to identify subpopulations with distinct trajectories of renal function and to identify factors discriminating these subpopulations. For example, Lemley *et al.* compared GFR courses over time between different groups of albuminuric patients using functional data analysis for longitudinal data [44]. O’Hare *et al.* studied trajectories of GFR using a latent class growth analysis, implemented in the SAS PROC TRAJ [45]. De Beaudrap *et al.* also used a latent class growth analysis but with the log of GFR as the outcome to achieve normality [9]. Briefly, the latent class growth analysis allows identification of classes of individuals following similar progressions of the outcome over time [46]. However, this method assumes no individual deviation from the class mean trajectory, and independence of repeated measurements of a same patient within the class [47]. An alternative method which does not have these limitations and has already been used in some contexts other than CKD is the latent class linear mixed model, also called growth mixture modelling [48-50]. Although this model is more complex to estimate than the more simple model in the latent class growth analysis, some statistical software packages such as Mplus [47] or the lcmm R package, are now available to perform such analyses (Table 4). The lcmm package also handles joint latent class models which may be used to account for informative censoring as discussed below [51].

##### Accounting for informative censoring-joint models

An important issue in longitudinal analyses of a quantitative marker of the renal function is early study dropout [52] due to, e.g. initiation of kidney replacement therapy. If the reason for dropout of the study is unrelated to renal function, the data missing after each individual last observed value of the marker are said to be “missing completely random” (MCAR). In this situation, both standard linear mixed models and standard linear GEE can be used to investigate the effect of risk factors on the mean trajectory of the renal function. If the reason for dropout is associated only with previously *observed* values of the marker (and not with *unobserved* values of the renal function after dropout), missing data due to dropout are said to be “missing at random” (MAR). In this situation, standard linear mixed models or a weighted version of GEE [53] can be used. However, if the reason for dropout is related to *non-observed* values of the renal function, missing data are said to be “non-random” or “informative”, and the dropout process should be jointly modelled with the marker of the renal function, or the analysis should be performed conditionally on the pattern of dropouts [54]. These approaches handling informative censoring should therefore be used to investigate risk factors of GFR trajectory if some patients dropout of the study because of kidney initiation therapy and one believes that initiation depends not only on previous observed values of GFR, but also on unobserved values of GFR. Out of the 91 occurrences where the outcome of interest was the quantitative measurement of the renal function over time, death or initiation of kidney replacement therapy (or other medical reasons) has been acknowledged as a source of informative censoring, and accounted for accordingly, on nine occasions (9.9%) (see e.g. [55-58]).

Different statistical approaches have been proposed to jointly model longitudinal quantitative markers and clinical events. They consist of considering a model for the renal function trajectory (usually a linear mixed model) and a model for the time-to-death or kidney replacement therapy (usually a Cox model), and linking both models using a shared latent structure. The most popular joint modelling approach uses a shared random-effects model. In a methodological paper, Vonesh *et al.* proposed such an approach to analyse data from the modification of diet in renal diseases (MDRD) study [58], and provided some SAS code necessary to implement the method (Table 4). Shared random-effects models are also implemented in the JM R package [59] (Table 4). An alternative approach consists in using a joint latent class model. As opposed to the first approach, the joint latent class model assumes that the population is divided into various subpopulations with different longitudinal evolutions of the quantitative marker associated with different risk functions for the event [51]. In the context of CKD, such an approach has been proposed by Garre *et al.* to jointly model the reciprocal of serum creatinine and time to renal graft failure [60]. Joint latent class models are implemented in the R package lcmm.

It should be noted that the joint modelling approach can also be used for other purposes including accounting for the trajectory of the quantitative marker to dynamically predict the clinical event [51].

## Conclusions

This paper provides an overview of the state of the art of statistical regression methods used to investigate risk factors of CKD outcomes. Although our review is not an exhaustive review of all statistical methods used in this context, we are confident that it highlights important statistical issues in studies of risk factors of CKD progression and discusses how they can be accounted for by using appropriate existing methods. It should be mentioned that because our aim was to provide an overview of regression methods for investigating risk factors, so for aetiological research, we did not focus our attention to studies aiming at establishing risk prediction models. However, some of the issues that we addressed in this paper also apply in the context of prediction of CKD outcomes. For example, competing risks should also be accounted for in this context [61], and new metrics for the evaluation of prognosis performance should be used [62].

As all overviews, our study is just an introduction to issues that would all merit further discussion. On the other hand, many of these issues are interrelated and only an overview can provide a picture of the connection between them. In particular, interval censoring complicates competing risks analyses of CKD progression, and specific methods accounting for both issues should be used, especially when investigating populations at relatively high risk of dying and when the time interval between consecutive measurements of the renal function may be long for some patients [24]. For longitudinal analyses where the outcome of interest is the whole trajectory of the renal function over time, we discussed the need to use linear mixed models or GEE to account for all the information available on the repeated measurements of the renal function for each patient, as well as to obtain reliable confidence intervals of risk factor effects on the renal trajectory. We also highlighted some methods to identify subpopulations with different trajectories of renal function over time, as well as methods to account for potential informative censoring due to death or kidney replacement therapy. Investigators have to be encouraged to account for these issues in statistical analyses, in order to obtain unbiased estimates of the effects of risk factors on CKD progression.

## Competing interests

The authors declare that they have no competing interests.

## Authors’ contributions

JB and KL conceived the idea and drafted the outline and paper. JB performed literature search and data abstraction. All authors reviewed several draft versions of the manuscript and approved the final manuscript.

## Pre-publication history

The pre-publication history for this paper can be accessed here:

http://www.biomedcentral.com/1471-2369/15/45/prepub

## Supplementary Material

Additional file 1 **Classification of all 304 papers according to outcomes and statistical methods used.** This additional file 1 contains all references identified in our literature review. All references were classified according to outcomes and statistical methods used. The additional file 1 contains the references of the 304 papers included in our literature review. The reference numbers range from 8 to 350, to keep exactly the same reference numbers as in the body text. Click here for file
