# Supplementary material for: Regression methods for investigating risk factors of chronic kidney disease outcomes: the state of the art
Source: BMC Nephrol. 2014 Mar 14;15:45. doi: 10.1186/1471-2369-15-45 (PMC4004351; doi:10.1186/1471-2369-15-45)
Supplement: Additional file 1 — Classification of all 304 papers according to outcomes and statistical methods used. This additional file 1 contains all references identified in our literature review. All references were classified according to outcomes and statistical methods used. The additional file 1 contains the references of the 304 papers included in our literature review. The reference numbers range from 8 to 350, to keep exactly the same reference numbers as in the body text. [file 1471-2369-15-45-S1.pdf]

Table 1: Classification of papers according to regression models (Cox, cause-specific, or Fine and Gray and logistic) used to investigate risk factors of time-to-event outcomes.

**Outcome investigated**	Regression model mentioned	References
**Exactly known time-to-event**		
All-cause death	Cox model	[13, 17, 36, 63-167]
	Cause-specific model	[8, 13, 79, 168-174]
	Fine and Gray model	[8, 14, 36, 69, 80, 152, 169, 171, 175, 176]
	Logistic model	[177-180]
Cardiovascular death	Cox model	[70, 72, 74, 77, 91, 93, 95-97, 115, 122, 124-129, 132, 135, 147, 151, 156, 164, 167, 181-185]
	Cause-specific model	[186]
	Fine and Gray model	[175]
Cardiovascular event	Cox model	[71, 78, 83, 95, 115, 135, 139, 148, 164, 165, 187-199]
Initiation of kidney replacement therapy or death due to kidney failure	Cox model	[64, 65, 68, 74, 76, 78, 81, 87-89, 91, 95-97, 102, 112, 114, 116, 118, 120, 130, 131, 140, 142, 143, 145, 152, 155, 161, 162, 188, 189, 200-232]
	Fine and Gray model	[8, 14, 69, 80, 136, 152, 169, 175, 176, 210]
	Cause-specific model	[8, 13, 79, 169, 172, 173, 228]
	Logistic model	[233]
Initiation of kidney replacement therapy or death (whichever comes first)	Cox model	[17, 37, 69, 74, 88, 90, 96, 97, 102, 103, 105, 106, 108, 112-114, 124, 129, 143, 167, 215, 217, 220, 225-227, 232, 234-244]
**Interval-censored time-to-event**		
Absolute or relative change in renal function higher than a specific value as compared to baseline value, based on<br>- GFR<br>- creatinine clearance<br>- proteinuria	Cox model<br>Fine and Gray model<br>Logistic model	[36, 163, 245-249]<br>[36]<br>[89, 169, 213, 250-257]
Transition to a specific stage of disease (based on CKD or proteinuria)	Cox model	[9, 92, 146, 235, 245, 258-261]
	Logistic model	[262-265]
Doubling of creatinine (serum or clearance)	Cox model<br>Logistic model	[130, 131, 211, 266-269]<br>[270]
**Composite of exact and interval-censored time-to-events**	Cox model	[36, 63, 78, 83, 141, 148, 153, 190, 192, 194, 201, 209, 211, 238, 271-291]
	Fine and Gray model	[36, 171, 292]
	Cause-specific model	[168, 170, 171]
	Logistic model	[179, 293]

Abbreviations: GFR, glomerular filtration rate; CKD, chronic kidney disease

Table 2: Classification of papers according to regression models (standard linear or linear mixed model, and generalized estimating equations) used to investigate repeated measurements of renal function.

Outcome investigated	Regression model mentioned	References
**All repeated measurements of renal function**		
Repeated measurements of<br>- GFR<br>- Creatinine clearance<br>- Proteinuria	Linear mixed model	[17, 36, 37, 41, 55, 56, 236, 292, 294-307]
	Linear mixed model accounting for informative drop-out	[55-58, 107, 170, 297, 308, 309]
	Linear GEE	[216, 291, 295, 310]
	Linear GEE accounting for informative drop-out	[311]
	Latent class growth analysis	[45]
Repeated measurements of<br>- log GFR	Linear mixed model	[9, 140, 260, 312-314]
- log creatinine (serum or clearance)	Linear GEE	[38, 84]
- log proteinuria	Latent class growth analysis	[9]
Absolute GFR change between each visit and baseline	Linear mixed model	[299]
Relative GFR change each year	Linear GEE	[163]
**A summary statistic for the change of renal function**		
Individual slope of<br>- GFR<br>- Creatinine (serum or clearance)<br>- UACR	Linear model	[37, 69, 96, 97, 118, 204, 224, 240, 246, 251, 252, 254, 267, 273, 278, 315-331]
Absolute GFR change as compared to baseline	Linear model	[81, 245, 255, 262, 332-334]
Relative GFR change as compared to baseline	Linear model	[81]
Log of absolute proteinuria change as compared to baseline	Linear model	[245]

Abbreviations: GFR, glomerular filtration rate; GEE, generalized estimating equations; UACR, urine albumin-to-creatinine ratio

[a] Slope of a marker is a summary statistic derived from measurements of a patient

Papers that investigated outcomes that are not mentioned in Table 1 and 2: [18, 29, 335-350].

8. Lim HJ, Zhang X, Dyck R, Osgood N: **Methods of Competing Risks Analysis of End-Stage Renal Disease and Mortality among People with Diabetes.** *BMC Med Res Methodol* 2010, **10:**97.
9. De Beaudrap P, Diallo MB, Landman R, Gueye NF, Ndiaye I, Diouf A, Kane CT, Etard JF, Girard PM, Sow PS, Delaporte E: **Changes in the renal function after tenofovir-containing antiretroviral therapy initiation in a Senegalese cohort (ANRS 1215).** *AIDS Res Hum Retroviruses* 2010, **26:**1221-1227.
13. De Nicola L, Minutolo R, Chiodini P, Zamboli P, Cianciaruso B, Nappi F, Signoriello S, Conte G, Zoccali C, Group S-TCS: **Prevalence and prognosis of mild anemia in non-dialysis chronic kidney disease: a prospective cohort study in outpatient renal clinics.** *Am J Nephrol* 2010, **32:**533-540.
14. Forsblom C, Harjutsalo V, Thorn LM, Waden J, Tolonen N, Saraheimo M, Gordin D, Moran JL, Thomas MC, Groop PH: **Competing-risk analysis of ESRD and death among patients with type 1 diabetes and macroalbuminuria.** *J Am Soc Nephrol* 2011, **22:**537-544.
17. Cianciaruso B, Pota A, Bellizzi V, Di Giuseppe D, Di Micco L, Minutolo R, Pisani A, Sabbatini M, Ravani P: **Effect of a low- versus moderate-protein diet on progression of CKD: follow-up of a randomized controlled trial.** *Am J Kidney Dis* 2009, **54:**1052-1061.
18. Scolari F, Ravani P, Gaggi R, Santostefano M, Rollino C, Stabellini N, Colla L, Viola BF, Maiorca P, Venturelli C, et al: **The challenge of diagnosing atheroembolic renal disease: clinical features and prognostic factors.** *Circulation* 2007, **116:**298-304.
29. Hu B, Li L, Wang X, Greene T: **Nonparametric multistate representations of survival and longitudinal data with measurement error.** *Stat Med* 2012, **31:**2303-2317.
36. Alves TP, Hulgan T, Wu P, Sterling TR, Stinnette SE, Rebeiro PF, Vincz AJ, Bruce M, Ikizler TA: **Race, kidney disease progression, and mortality risk in HIV-infected persons.** *Clin J Am Soc Nephrol* 2010, **5:**2269-2275.
37. Evans M, Fored CM, Bellocco R, Fitzmaurice G, Fryzek JP, McLaughlin JK, Nyren O, Elinder CG: **Acetaminophen, aspirin and progression of advanced chronic kidney disease.** *Nephrol Dial Transplant* 2009, **24:**1908-1918.
38. Agarwal M, Selvan V, Freedman BI, Liu Y, Wagenknecht LE: **The relationship between albuminuria and hormone therapy in postmenopausal women.** *Am J Kidney Dis* 2005, **45:**1019-1025.
41. Ishwaran H, Takahara G: **Independent and identically distributed Monte Carlo algorithms for semiparametric linear mixed models.** *J Am Stat Assoc* 2002, **97:**1154-1166.
45. O'Hare AM, Batten A, Burrows NR, Pavkov ME, Taylor L, Gupta I, Todd-Stenberg J, Maynard C, Rodriguez RA, Murtagh FE, et al: **Trajectories of kidney function decline in the 2 years before initiation of long-term dialysis.** *Am J Kidney Dis* 2012, **59:**513-522.
55. Eriksen BO, Tomtum J, Ingebretsen OC: **Predictors of declining glomerular filtration rate in a population-based chronic kidney disease cohort.** *Nephron Clin Pract* 2010, **115:**c41-50.
56. Fung MM, Salem RM, Lipkowitz MS, Bhatnagar V, Pandey B, Schork NJ, O'Connor DT: **Methylenetetrahydrofolate reductase (MTHFR) polymorphism A1298C (Glu429Ala) predicts decline in renal function over time in the African-American Study of Kidney Disease and Hypertension (AASK) Trial and Veterans Affairs Hypertension Cohort (VAHC).** *Nephrol Dial Transplant* 2012, **27:**197-205.
57. Li J, Schluchter MD: **Conditional mixed models adjusting for non-ignorable drop-out with administrative censoring in longitudinal studies.** *Stat Med* 2004, **23:**3489-3503.
58. Vonesh EF, Greene T, Schluchter MD: **Shared parameter models for the joint analysis of longitudinal data and event times.** *Stat Med* 2006, **25:**143-163.
63. Agarwal R: **Blood pressure components and the risk for end-stage renal disease and death in chronic kidney disease.** *Clin J Am Soc Nephrol* 2009, **4:**830-837.
64. Agarwal R, Debella YT, Giduma HD, Light RP: **Long-term retinal, renal and cardiovascular outcomes in diabetic chronic kidney disease without proteinuria.** *Nephrol Dial Transplant* 2012, **27:**310-317.
65. Agarwal R, Kariyanna SS, Light RP: **Prognostic value of circadian blood pressure variation in chronic kidney disease.** *Am J Nephrol* 2009, **30:**547-553.
66. Ahmed A, Love TE, Sui X, Rich MW: **Effects of Angiotensin-Converting Enzyme Inhibitors in Systolic Heart Failure Patients With Chronic Kidney Disease: A Propensity Score Analysis.** *J Card Fail* 2006, **12:**499-506.

67. Al-Aly Z, Zeringue A, Fu J, Rauchman MI, McDonald JR, El-Achkar TM, Balasubramanian S, Nurutdinova D, Xian H, Stroupe K, et al: **Rate of kidney function decline associates with mortality.** *J Am Soc Nephrol* 2010, **21:**1961-1969.
68. Astor BC, Matsushita K, Gansevoort RT, Van Der Velde M, Woodward M, Levey AS, Jong PED, Coresh J, El-Nahas M, Eckardt KU, et al: **Lower estimated glomerular filtration rate and higher albuminuria are associated with mortality and end-stage renal disease. A collaborative meta-analysis of kidney disease population cohorts.** *Kidney Int* 2011, **79:**1331-1340.
69. Barbour SJ, Er L, Djurdjev O, Karim MM, Levin A: **Differences in progression of CKD and mortality amongst Caucasian, Oriental Asian and South Asian CKD patients.** *Nephrol Dial Transplant* 2010, **25:**3663-3672.
70. Barreto FC, Barreto DV, Liabeuf S, Meert N, Glorieux G, Temmar M, Choukroun G, Vanholder R, Massy ZA: **Serum indoxyl sulfate is associated with vascular disease and mortality in chronic kidney disease patients.** *Clin J Am Soc Nephrol* 2009, **4:**1551-1558.
71. Beddhu S, Nigwekar SU, Ma X, Greene T: **Associations of resting heart rate with insulin resistance, cardiovascular events and mortality in chronic kidney disease.** *Nephrol Dial Transplant* 2009, **24:**2482-2488.
72. Bowling CB, Pitt B, Ahmed MI, Aban IB, Sanders PW, Mujib M, Campbell RC, Love TE, Aronow WS, Allman RM, et al: **Hypokalemia and outcomes in patients with chronic heart failure and chronic kidney disease findings from propensity-matched studies.** *Circ Heart Fail* 2010, **3:**253-260.
73. Breton G, Froissart M, Janus N, Launay-Vacher V, Berr C, Tzourio C, Helmer C, Stengel B: **Inappropriate drug use and mortality in community-dwelling elderly with impaired kidney function-the Three-City population-based study.** *Nephrol Dial Transplant* 2011, **26:**2852-2859.
74. Chawla V, Greene T, Beck GJ, Kusek JW, Collins AJ, Sarnak MJ, Menon V: **Hyperlipidemia and long-term outcomes in nondiabetic chronic kidney disease.** *Clin J Am Soc Nephrol* 2010, **5:**1582-1587.
75. Chen JLT, Lerner D, Ruthazer R, Castaneda-Sceppa C, Levey AS: **Association of physical activity with mortality in chronic kidney disease.** *J Nephrol* 2008, **21:**243-252.
76. Chiu YL, Chien KL, Lin SL, Chen YM, Tsai TJ, Wu KD: **Outcomes of stage 3-5 chronic kidney disease before end-stage renal disease at a single center in Taiwan.** *Nephron Clin Pract* 2008, **109:**c109-c118.
77. Chronic Kidney Disease Prognosis Consortium, Matsushita K, van der Velde M, Astor BC, Woodward M, Levey AS, de Jong PE, Coresh J, Gansevoort RT: **Association of estimated glomerular filtration rate and albuminuria with all-cause and cardiovascular mortality in general population cohorts: a collaborative meta-analysis.** *Lancet* 2010, **375:**2073-2081.
78. Clase CM, Gao P, Tobe SW, McQueen MJ, Grosshennig A, Teo KK, Yusuf S, Mann JFE: **Estimated glomerular filtration rate and albuminuria as predictors of outcomes in patients with high cardiovascular risk: A cohort study.** *Ann Intern Med* 2011, **154:**310-318.
79. De Nicola L, Chiodini P, Zoccali C, Borrelli S, Cianciaruso B, Lorio BD, Santoro D, Giancaspro V, Abaterusso C, Gallo C, et al: **Prognosis of CKD patients receiving outpatient nephrology care in italy.** *Clin J Am Soc Nephrol* 2011, **6:**2421-2428.
80. De Nicola L, Minutolo R, Chiodini P, Borrelli S, Zoccali C, Postorino M, Iodice C, Nappi F, Fuiano G, Gallo C, Conte G: **The effect of increasing age on the prognosis of non-dialysis patients with chronic kidney disease receiving stable nephrology care.** *Kidney Int* 2012, **82:**482-488.
81. Dichtel LE, Gurevich DF, Rifkin B, Varma P, Concato J, Peixoto AJ: **Renal artery revascularization in patients with atherosclerotic renal artery stenosis and impaired renal function: Conservative management versus renal artery stenting.** *Clin Nephrol* 2010, **74:**113-122.
82. Eddington H, Hoefield R, Sinha S, Chrysochou C, Lane B, Foley RN, Hegarty J, New J, O'Donoghue DJ, Middleton RJ, Kalra PA: **Serum phosphate and mortality in patients with chronic kidney disease.** *Clin J Am Soc Nephrol* 2010, **5:**2251-2257.
83. Fischer MJ, Kimmel PL, Greene T, Gassman JJ, Wang X, Brooks DH, Charleston J, Dowie D, Thornley-Brown D, Cooper LA, et al: **Elevated depressive affect is associated with adverse cardiovascular outcomes among African Americans with chronic kidney disease.** *Kidney Int* 2011, **80:**670-678.
84. Gardner LI, Holmberg SD, Williamson JM, Szczech LA, Carpenter CCJ, Rompalo AM, Schuman P, Klein RS: **Development of proteinuria or elevated serum creatinine and mortality in HIV-infected women.** *J Acquir Immune Defic Syndr* 2003, **32:**203-209.
85. Goicoechea M, de Vinuesa SG, Gómez-Campderá F, Aragoncillo I, Verdalles U, Mosse A, Luño J: **Serum fibrinogen levels are an independent predictor of mortality in patients with chronic kidney disease (CKD) stages 3 and 4.** *Kidney Int Suppl* 2008:S67-70.
86. Hartle JE, Tang X, Kirchner HL, Bucaloiu ID, Sartorius JA, Pogrebnaya ZV, Akers GA, Carnero GE, Perkins RM: **Bisphosphonate therapy, death, and cardiovascular events among female patients with CKD: A retrospective cohort study.** *Am J Kidney Dis* 2012, **59:**636-644.

87. Haynes R, Hutchison CA, Emberson J, Dasgupta T, Wheeler DC, Townend JN, Landray MJ, Cockwell P: **Serum free light chains and the risk of ESRD and death in CKD.** *Clin J Am Soc Nephrol* 2011, **6:**2829-2837.
88. Hoefield RA, Kalra PA, Baker P, Lane B, New JP, O'Donoghue DJ, Foley RN, Middleton RJ: **Factors associated with kidney disease progression and mortality in a referred CKD population.** *Am J Kidney Dis* 2010, **56:**1072-1081.
89. Hossain MP, Palmer D, Goyder E, El Nahas AM: **Association of deprivation with worse outcomes in chronic kidney disease: Findings from a Hospital-Based Cohort in the United Kingdom.** *Nephron Clin Pract* 2012, **120:**c59-c70.
90. Hovind P, Tarnow L, Rossing P, Carstensen B, Parving HH: **Improved survival in patients obtaining remission of nephrotic range albuminuria in diabetic nephropathy.** *Kidney Int* 2004, **66:**1180-1186.
91. Hwang SJ, Lin MY, Chen HC, Hwang SC, Yang WC, Hsu CC, Chiu HC, Mau LW: **Increased risk of mortality in the elderly population with late-stage chronic kidney disease: A cohort study in Taiwan.** *Nephrol Dial Transplant* 2008, **23:**3192-3198.
92. Ishani A, Nelson D, Clothier B, Schult T, Nugent S, Greer N, Slinin Y, Ensrud KE: **The magnitude of acute serum creatinine increase after cardiac surgery and the risk of chronic kidney disease, progression of kidney disease, and death.** *Arch Intern Med* 2011, **171:**226-233.
93. Ix JH, Shlipak MG, Sarnak MJ, Beck GJ, Greene T, Wang X, Kusek JW, Collins AJ, Levey AS, Menon V: **Fetuin-A is not associated with mortality in chronic kidney disease.** *Kidney Int* 2007, **72:**1394-1399.
94. Jamison RL, Hartigan P, Kaufman JS, Goldfarb DS, Warren SR, Guarino PD, Gaziano JM: **Effect of homocysteine lowering on mortality and vascular disease in advanced chronic kidney disease and end-stage renal disease: A randomized controlled trial.** *JAMA* 2007, **298:**1163-1170.
95. Jardine MJ, Ninomiya T, Perkovic V, Cass A, Turnbull F, Gallagher MP, Zoungas S, Lambers Heerspink HJ, Chalmers J, Zanchetti A: **Aspirin is beneficial in hypertensive patients with chronic kidney disease: A post-hoc subgroup analysis of a randomized controlled trial.** *J Am Coll Cardiol* 2010, **56:**956-965.
96. Jorsal A, Tarnow L, Frystyk J, Lajer M, Flyvbjerg A, Parving HH, Vionnet N, Rossing P: **Serum adiponectin predicts all-cause mortality and end stage renal disease in patients with type I diabetes and diabetic nephropathy.** *Kidney Int* 2008, **74:**649-654.
97. Jorsal A, Tarnow L, Lajer M, Ek J, Hansen T, Pedersen O, Parving HH: **The PPARγ2 Pro12Ala variant predicts ESRD and mortality in patients with type 1 diabetes and diabetic nephropathy.** *Mol Genet Metab* 2008, **94:**347-351.
98. Kalra PA, Chrysochou C, Green D, Cheung CM, Khavandi K, Sixt S, Rastan A, Zeller T: **The benefit of renal artery stenting in patients with atheromatous renovascular disease and advanced chronic kidney disease.** *Catheter Cardiovasc Interv* 2010, **75:**1-10.
99. Kellerman QD, Christensen AJ, Baldwin AS, Lawton WJ: **Association between depressive symptoms and mortality risk in chronic kidney disease.** *Health Psychol* 2010, **29:**594-600.
100. Kestenbaum B, Sampson JN, Rudser KD, Patterson DJ, Seliger SL, Young B, Sherrard DJ, Andress DL: **Serum phosphate levels and mortality risk among people with chronic kidney disease.** *J Am Soc Nephrol* 2005, **16:**520-528.
101. Khan F, Adelstein E, Saba S: **Implantable cardioverter defibrillators confer survival benefit in patients with renal insufficiency but not in dialysis-dependent patients.** *J Interv Card Electrophysiol* 2010, **28:**117-123.
102. Korgaonkar S, Tilea A, Gillespie BW, Kiser M, Eisele G, Finkelstein F, Kotanko P, Pitt B, Saran R: **Serum potassium and outcomes in CKD: Insights from the RRI-CKD cohort study.** *Clin J Am Soc Nephrol* 2010, **5:**762-769.
103. Kovesdy CP, Ahmadzadeh S, Anderson JE, Kalantar-Zadeh K: **Secondary hyperparathyroidism is associated with higher mortality in men with moderate to severe chronic kidney disease.** *Kidney Int* 2008, **73:**1296-1302.
104. Kovesdy CP, Anderson JE, Kalantar-Zadeh K: **Paradoxical Association Between Body Mass Index and Mortality in Men With CKD Not Yet on Dialysis.** *Am J Kidney Dis* 2007, **49:**581-591.
105. Kovesdy CP, Anderson JE, Kalantar-Zadeh K: **Association of serum bicarbonate levels with mortality in patients with non-dialysis-dependent CKD.** *Nephrol Dial Transplant* 2009, **24:**1232-1237.
106. Kovesdy CP, Anderson JE, Kalantar-Zadeh K: **Outcomes associated with serum phosphorus level in males with non-dialysis dependent chronic kidney disease.** *Clin Nephrol* 2010, **73:**268-275.

107. Kovesdy CP, Kuchmak O, Lu JL, Kalantar-Zadeh K: **Outcomes associated with phosphorus binders in men with non-dialysis-dependent CKD.** *Am J Kidney Dis* 2010, **56:**842-851.
108. Kovesdy CP, Kuchmak O, Lu JL, Kalantar-Zadeh K: **Outcomes associated with serum calcium level in men with non-dialysis-dependent chronic kidney disease.** *Clin J Am Soc Nephrol* 2010, **5:**468-476.
109. Kovesdy CP, Lott EH, Lu JL, Malakauskas SM, Ma JZ, Molnar MZ, Kalantar-Zadeh K: **Hyponatremia, hypernatremia, and mortality in patients with chronic kidney disease with and without congestive heart failure.** *Circulation* 2012, **125:**677-684.
110. Kovesdy CP, Trivedi BK, Anderson JE: **Association of kidney function with mortality in patients with chronic kidney disease not yet on dialysis: A historical prospective cohort study.** *Adv Chronic Kidney Dis* 2006, **13:**183-188.
111. Kovesdy CP, Trivedi BK, Kalantar-Zadeh K, Anderson JE: **Association of low blood pressure with increased mortality in patients with moderate to severe chronic kidney disease.** *Nephrol Dial Transplant* 2006, **21:**1257-1262.
112. Kovesdy CP, Trivedi BK, Kalantar-Zadeh K, Anderson JE: **Association of anemia with outcomes in men with moderate and severe chronic kidney disease.** *Kidney Int* 2006, **69:**560-564.
113. Kovesdy CP, Ureche V, Lu JL, Kalantar-Zadeh K: **Outcome predictability of serum alkaline phosphatase in men with pre-dialysis CKD.** *Nephrol Dial Transplant* 2010, **25:**3003-3011.
114. Kumar B, Tilea A, Gillespie BW, Zhang X, Kiser M, Eisele G, Finkelstein F, Kotanko P, Levin N, Rajagopalan S, Saran R: **Significance of self-reported sleep quality (SQ) in chronic kidney disease (CKD): The Renal Research Institute (RRI)-CKD study.** *Clin Nephrol* 2010, **73:**104-114.
115. Lambers Heerspink HJ, Ninomiya T, Perkovic V, Woodward M, Zoungas S, Cass A, Cooper M, Grobbee DE, Mancia G, Mogensen CE, et al: **Effects of a fixed combination of perindopril and indapamide in patients with type 2 diabetes and chronic kidney disease.** *Eur Heart J* 2010, **31:**2888-2896.
116. Landray MJ, Emberson JR, Blackwell L, Dasgupta T, Zakeri R, Morgan MD, Ferro CJ, Vickery S, Ayrton P, Nair D, et al: **Prediction of ESRD and death among people with CKD: The chronic renal impairment in Birmingham (CRIB) prospective cohort study.** *Am J Kidney Dis* 2010, **56:**1082-1094.
117. Lee PH, Wu VC, Hu FC, Lai CF, Chen YM, Tsai TJ, Wu KD: **Outcomes following dialysis for acute kidney injury among different stages of chronic kidney disease.** *Am J Nephrol* 2011, **34:**95-103.
118. Levin A, Djurdjev O, Beaulieu M, Er L: **Variability and Risk Factors for Kidney Disease Progression and Death Following Attainment of Stage 4 CKD in a Referred Cohort.** *Am J Kidney Dis* 2008, **52:**661-671.
119. Levin A, Djurdjev O, Duncan J, Rosenbaum D, Werb R: **Haemoglobin at time of referral prior to dialysis predicts survival: An association of haemoglobin with long-term outcomes.** *Nephrol Dial Transplant* 2006, **21:**370-377.
120. Li S, Foley RN, Collins AJ: **Anemia and cardiovascular disease, hospitalization, end stage renal disease, and death in older patients with chronic kidney disease.** *Int Urol Nephrol* 2005, **37:**395-402.
121. Liabeuf S, Barreto DV, Barreto FC, Chasseraud M, Brazier M, Choukroun G, Kamel S, Massy ZA: **The circulating soluble TRAIL is a negative marker for inflammation inversely associated with the mortality risk in chronic kidney disease patients.** *Nephrol Dial Transplant* 2010, **25:**2596-2602.
122. Liabeuf S, Barreto DV, Kretschmer A, Barreto FC, Renard C, Andrejak M, Choukroun G, Massy Z: **High circulating levels of large splice variants of tenascin-C is associated with mortality and cardiovascular disease in chronic kidney disease patients.** *Atherosclerosis* 2011, **215:**116-124.
123. Lu TM, Chung MY, Lin CC, Hsu CP, Lin SJ: **Asymmetric dimethylarginine and clinical outcomes in chronic kidney disease.** *Clin J Am Soc Nephrol* 2011, **6:**1566-1572.
124. Madero M, Sarnak MJ, Wang X, Sceppa CC, Greene T, Beck GJ, Kusek JW, Collins AJ, Levey AS, Menon V: **Body Mass Index and Mortality in CKD.** *Am J Kidney Dis* 2007, **50:**404-411.
125. Meisinger C, Döring A, Löwel H: **Chronic kidney disease and risk of incident myocardial infarction and all-cause and cardiovascular disease mortality in middle-aged men and women from the general population.** *Eur Heart J* 2006, **27:**1245-1250.
126. Menon V, Greene T, Pereira AA, Wang X, Beck GJ, Kusek JW, Collins AJ, Levey AS, Sarnak MJ: **Glycosylated hemoglobin and mortality in patients with nondiabetic chronic kidney disease.** *J Am Soc Nephrol* 2005, **16:**3411-3417.

127. Menon V, Greene T, Pereira AA, Wang X, Beck GJ, Kusek JW, Collins AJ, Levey AS, Sarnak MJ: **Relationship of phosphorus and calcium-phosphorus product with mortality in CKD.** *Am J Kidney Dis* 2005, **46:**455-463.
128. Menon V, Greene T, Wang X, Pereira AA, Marcovina SM, Beck GJ, Kusek JW, Collins AJ, Levey AS, Sarnak MJ: **C-reactive protein and albumin as predictors of all-cause and cardiovascular mortality in chronic kidney disease.** *Kidney Int* 2005, **68:**766-772.
129. Menon V, Li L, Wang X, Greene T, Balakrishnan V, Madero M, Pereira AA, Beck GJ, Kusek JW, Collins AJ, et al: **Adiponectin and mortality in patients with chronic kidney disease.** *J Am Soc Nephrol* 2006, **17:**2599-2606.
130. Methven S, MacGregor MS, Traynor JP, Hair M, O'Reilly DSJ, Deighan CJ: **Comparison of urinary albumin and urinary total protein as predictors of patient outcomes in CKD.** *Am J Kidney Dis* 2011, **57:**21-28.
131. Methven S, Traynor JP, Hair MD, St J O'Reilly D, Deighan CJ, MacGregor MS: **Stratifying risk in chronic kidney disease: an observational study of UK guidelines for measuring total proteinuria and albuminuria.** *QJM* 2011, **104:**663-670.
132. Miyamoto T, Carrero JJ, Qureshi AR, Anderstam B, Heimbürger O, Bárány P, Lindholm B, Stenvinkel P: **Circulating follistatin in patients with chronic kidney disease: Implications for muscle strength, bone mineral density, inflammation, and survival.** *Clin J Am Soc Nephrol* 2011, **6:**1001-1008.
133. Murussi M, Campagnolo N, Beck MO, Gross JL, Silveiro SP: **High-normal levels of albuminuria predict the development of micro- and macroalbuminuria and increased mortality in Brazilian Type 2 diabetic patients: An 8-year follow-up study.** *Diabet Med* 2007, **24:**1136-1142.
134. Neves PL, Malho A, Cabrita A, Pinho A, Baptista A, Morgado E, Faísca M, Carrasqueira H, Silva AP: **Statins and vitamin D: A friendly association in pre-dialysis patients.** *Int Urol Nephrol* 2010, **42:**173-179.
135. Norris K, Bourgoigne J, Gassman J, Hebert L, Middleton J, Phillips RA, Randall O, Rostand S, Sherer S, Toto RD, et al: **Cardiovascular Outcomes in the African American Study of Kidney Disease and Hypertension (AASK) Trial.** *Am J Kidney Dis* 2006, **48:**739-751.
136. Obi Y, Kimura T, Nagasawa Y, Yamamoto R, Yasuda K, Sasaki K, Kitamura H, Imai E, Rakugi H, Isaka Y, Hayashi T: **Impact of age and overt proteinuria on outcomes of stage 3 to 5 chronic kidney disease in a referred cohort.** *Clin J Am Soc Nephrol* 2010, **5:**1558-1565.
137. Perkins RM, Bucaloiu ID, Kirchner HL, Ashouian N, Hartle JE, Yahya T: **GFR decline and mortality risk among patients with chronic kidney disease.** *Clin J Am Soc Nephrol* 2011, **6:**1879-1886.
138. Ramachandran J, Juneja R, John L, Dutta AK, Chen JW, Woodman RJ, Wigg AJ: **Chronic kidney disease following liver transplantation: A south australian experience.** *Transplant Proc* 2010, **42:**3644-3646.
139. Ramkumar N, Murtaugh MA, Cheung AK, Beddhu S: **Lack of Synergistic Effects of Metabolic Syndrome and Plasma Fibrinogen on Coronary Events and Mortality in Moderate CKD.** *Am J Kidney Dis* 2007, **49:**356-364.
140. Reed B, Helal I, McFann K, Wang W, Yan XD, Schrier RW: **The impact of type II diabetes mellitus in patients with autosomal dominant polycystic kidney disease.** *Nephrol Dial Transplant* 2012, **27:**2862-2865.
141. Rossing K, Christensen PK, Hovind P, Parving HH: **Remission of nephrotic-range albuminuria reduces risk of end-stage renal disease and improves survival in type 2 diabetic patients.** *Diabetologia* 2005, **48:**2241-2247.
142. Sandvik MK, Iversen BM, Irgens LM, Skjaerven R, Leivestad T, Søfteland E, Vikse BE: **Are adverse pregnancy outcomes risk factors for development of end-stage renal disease in women with diabetes?** *Nephrol Dial Transplant* 2010, **25:**3600-3607.
143. Schumock GT, Andress D, Marx SE, Sterz R, Joyce AT, Kalantar-Zadeh K: **Impact of secondary hyperparathyroidism on disease progression, healthcare resource utilization and costs in pre-dialysis CKD patients.** *Curr Med Res Opin* 2008, **24:**3037-3048.
144. Schurgers LJ, Barreto DV, Barreto FC, Liabeuf S, Renard C, Magdeleyns EJ, Vermeer C, Choukroun G, Massy ZA: **The circulating inactive form of matrix gla protein is a surrogate marker for vascular calcification in chronic kidney disease: A preliminary report.** *Clin J Am Soc Nephrol* 2010, **5:**568-575.
145. Scolari F, Ravani P, Pola A, Guerini S, Zubani R, Movilli E, Savoldi S, Malberti F, Maiorca R: **Predictors of renal and patient outcomes in atheroembolic renal disease: A prospective study.** *J Am Soc Nephrol* 2003, **14:**1584-1590.

146. Senevirathna L, Abeysekera T, Nanayakkara S, Chandrajith R, Ratnatunga N, Harada KH, Hitomi T, Komiya T, Muso E, Koizumi A: **Risk factors associated with disease progression and mortality in chronic kidney disease of uncertain etiology: A cohort study in Medawachchiya, Sri Lanka.** *Environ Health Prev Med* 2012, **17:**191-198.
147. Sheng X, Murphy MJ, Macdonald TM, Wei L: **Effectiveness of statins in chronic kidney disease.** *QJM* 2012, **105:**641-648.
148. Shurraw S, Hemmelgarn B, Lin M, Majumdar SR, Klarenbach S, Manns B, Bello A, James M, Turin TC, Tonelli M: **Association between glycemic control and adverse outcomes in people with diabetes mellitus and chronic kidney disease: A population-based cohort study.** *Arch Intern Med* 2011, **171:**1920-1927.
149. Smith ER, Tomlinson LA, Ford ML, McMahon LP, Rajkumar C, Holt SG: **Elastin degradation is associated with progressive aortic stiffening and all-cause mortality in predialysis chronic kidney disease.** *Hypertension* 2012, **59:**973-978.
150. Solomon SD, Uno H, Lewis EF, Eckardt KU, Lin J, Burdmann EA, De Zeeuw D, Ivanovich P, Levey AS, Parfrey P, et al: **Erythropoietic response and outcomes in kidney disease and type 2 diabetes.** *N Engl J Med* 2010, **363:**1146-1155.
151. Sugiura S, Inaguma D, Kitagawa A, Murata M, Kamimura Y, Sendo S, Hamaguchi K, Nagaya H, Tatematsu M, Kurata K, et al: **Administration of alfacalcidol for patients with predialysis chronic kidney disease may reduce cardiovascular disease events.** *Clin Exp Nephrol* 2010, **14:**43-50.
152. Thomas MC, Moran J, Forsblom C, Harjutsalo V, Thorn L, Ahola A, Wadén J, Tolonen N, Saraheimo M, Gordin D, Groop PH: **The association between dietary sodium intake, ESRD, and all-cause mortality in patients with type 1 diabetes.** *Diabetes Care* 2011, **34:**861-866.
153. Thorp ML, Johnson ES, Yang X, Petrik AF, Platt R, Smith DH: **Effect of anaemia on mortality, cardiovascular hospitalizations and end-stage renal disease among patients with chronic kidney disease.** *Nephrology (Carlton)* 2009, **14:**240-246.
154. Turin TC, Coresh J, Tonelli M, Stevens PE, De Jong PE, Farmer CKT, Matsushita K, Hemmelgarn BR: **One-year change in kidney function is associated with an increased mortality risk.** *Am J Nephrol* 2012, **36:**41-49.
155. Uchigata Y, Asao K, Matsushima M, Sato A, Yokoyama H, Otani T, Kasahara T, Takaike H, Okudaira M, Miura J, et al: **Impact on mortality and incidence of end-stage renal disease of education and treatment at a diabetes center among patients with type 1 diabetes: Comparison of two subgroups in the Japanese DERI cohort.** *J Diabetes Complications* 2004, **18:**155-159.
156. Wang Y, Guo X, Li J, Hu D, Zhao D, Ma H, Mou Q, Liu J, Xu Y: **Predictive value of ankle-brachial index to all-cause mortality and cardiovascular mortality in Chinese patients with chronic kidney disease.** *Vasa* 2012, **41:**205-213.
157. Warnock DG, Muntner P, McCullough PA, Zhang X, McClure LA, Zakai N, Cushman M, Newsome BB, Kewalramani R, Steffes MW, et al: **Kidney function, albuminuria, and all-cause mortality in the REGARDS (Reasons for Geographic and Racial Differences in Stroke) study.** *Am J Kidney Dis* 2010, **56:**861-871.
158. Weber C, Sigrist M, Romann A, Chiarelli G, Levin A: **Novel biomarkers do not correlate with severity of vascular stiffness in ckd patients with severe co-morbid disease.** *Nephron Clin Pract* 2011, **119:**c261-c268.
159. Weiss JW, Johnson ES, Petrik A, Smith DH, Yang X, Thorp ML: **Systolic blood pressure and mortality among older community-dwelling adults with CKD.** *Am J Kidney Dis* 2010, **56:**1062-1071.
160. Wu IW, Hsu KH, Lee CC, Sun CY, Hsu HJ, Tsai CJ, Tzen CY, Wang YC, Lin CY, Wu MS: **P-cresyl sulphate and indoxyl sulphate predict progression of chronic kidney disease.** *Nephrol Dial Transplant* 2011, **26:**938-947.
161. Wu IW, Wang SY, Hsu KH, Lee CC, Sun CY, Tsai CJ, Wu MS: **Multidisciplinary predialysis education decreases the incidence of dialysis and reduces mortality-a controlled cohort study based on the NKFDOQI guidelines.** *Nephrol Dial Transplant* 2009, **24:**3426-3433.
162. Xue JL, Eggers PW, Agodoa LY, Foley RN, Collins AJ: **Longitudinal study of racial and ethnic differences in developing end-stage renal disease among aged medicare beneficiaries.** *J Am Soc Nephrol* 2007, **18:**1299-1306.
163. Yamashita T, Yoshida T, Ogawa T, Tsuchiya K, Nitta K: **Clinical outcomes in patients with chronic kidney disease: A 5-year retrospective cohort study at a University Hospital in Japan.** *Clin Exp Nephrol* 2011, **15:**831-840.
164. Yang JW, Han ST, Song SH, Kim MK, Kim JS, Choi SO, Han BG: **Serum T3 level can predict cardiovascular events and all-cause mortality rates in CKD patients with proteinuria.** *Ren Fail* 2012, **34:**364-372.

165. Yilmaz MI, Stenvinkel P, Sonmez A, Saglam M, Yaman H, Kilic S, Eyileten T, Caglar K, Oguz Y, Vural A, et al: **Vascular health, systemic inflammation and progressive reduction in kidney function; Clinical determinants and impact on cardiovascular outcomes.** *Nephrol Dial Transplant* 2011, **26:**3537-3543.
166. Young BA, Von Korff M, Heckbert SR, Ludman EJ, Rutter C, Lin EHB, Ciechanowski PS, Oliver M, Williams L, Himmelfarb J, Katon WJ: **Association of major depression and mortality in Stage 5 diabetic chronic kidney disease.** *Gen Hosp Psychiatry* 2010, **32:**119-124.
167. Young JM, Terrin N, Wang X, Greene T, Beck GJ, Kusek JW, Collins AJ, Sarnak MJ, Menon V: **Asymmetric dimethylarginine and mortality in stages 3 to 4 chronic kidney disease.** *Clin J Am Soc Nephrol* 2009, **4:**1115-1120.
168. Agarwal R, Bunaye Z, Bekele DM, Light RP: **Competing risk factor analysis of end-stage renal disease and mortality in chronic kidney disease.** *Am J Nephrol* 2008, **28:**569-575.
169. El-Ghoul B, Elie C, Sqalli T, Jungers P, Daudon M, Grünfeld JP, Lesavre P, Joly D: **Nonprogressive kidney dysfunction and outcomes in older adults with chronic kidney disease.** *J Am Geriatr Soc* 2009, **57:**2217-2223.
170. Eriksen BO, Ingebretsen OC: **The progression of chronic kidney disease: A 10-year population-based study of the effects of gender and age.** *Kidney Int* 2006, **69:**375-382.
171. Inrig JK, Barnhart HX, Reddan D, Patel UD, Sapp S, Califf RM, Singh AK, Szczech LA: **Effect of hemoglobin target on progression of kidney disease: A secondary analysis of the CHOIR (Correction of Hemoglobin and Outcomes in Renal Insufficiency) trial.** *Am J Kidney Dis* 2012, **60:**390-401.
172. Newsome BB, McClellan WM, Allison JJ, Eggers PW, Chen SC, Collins AJ, Kiefe CI, Coffey CS, Warnock DG: **Racial Differences in the Competing Risks of Mortality and ESRD After Acute Myocardial Infarction.** *Am J Kidney Dis* 2008, **52:**251-261.
173. Niewczas MA, Gohda T, Skupien J, Smiles AM, Walker WH, Rosetti F, Cullere X, Eckfeldt JH, Doria A, Mayadas TN, et al: **Circulating TNF receptors 1 and 2 predict ESRD in type 2 diabetes.** *J Am Soc Nephrol* 2012, **23:**507-515.
174. Ravani P, Tripepi G, Malberti F, Testa S, Mallamaci F, Zoccali C: **Asymmetrical dimethylarginine predicts progression to dialysis and death in patients with chronic kidney disease: A competing risks modeling approach.** *J Am Soc Nephrol* 2005, **16:**2449-2455.
175. Dalrymple LS, Katz R, Kestenbaum B, Shlipak MG, Sarnak MJ, Stehman-Breen C, Seliger S, Siscovick D, Newman AB, Fried L: **Chronic kidney disease and the risk of end-stage renal disease versus death.** *J Gen Intern Med* 2011, **26:**379-385.
176. Derose SF, Rutkowski MP, Levin NW, Liu ILA, Shi JM, Jacobsen SJ, Crooks PW: **Incidence of end-stage renal disease and death among insured African Americans with chronic kidney disease.** *Kidney Int* 2009, **76:**629-637.
177. Gullion CM, Keith DS, Nichols GA, Smith DH: **Impact of Comorbidities on Mortality in Managed Care Patients With CKD.** *Am J Kidney Dis* 2006, **48:**212-220.
178. Nakayama M, Sato T, Sato H, Yamaguchi Y, Obara K, Kurihara I, Sato K, Hotta O, Seino J, Miyata M, et al: **Different clinical outcomes for cardiovascular events and mortality in chronic kidney disease according to underlying renal disease: The Gonryo study.** *Clin Exp Nephrol* 2010, **14:**333-339.
179. Rucker D, Hemmelgarn BR, Lin M, Manns BJ, Klarenbach SW, Ayyalasomayajula B, James MT, Bello A, Gordon D, Jindal KK, Tonelli M: **Quality of care and mortality are worse in chronic kidney disease patients living in remote areas.** *Kidney Int* 2011, **79:**210-217.
180. Wang F, Zhang L, Zuo L, Liu L, Wang H: **Mortality and renal function decline among a community-based Chinese population with normal or mildly impaired renal function.** *Nephrol Dial Transplant* 2011, **26:**2847-2852.
181. Busch M, Fleck C, Wolf G, Stein G: **Asymmetrical (ADMA) and symmetrical dimethylarginine (SDMA) as potential risk factors for cardiovascular and renal outcome in chronic kidney disease - Possible candidates for paradoxical epidemiology?** *Amino Acids* 2006, **30:**225-232.
182. Elsayed EF, Tighiouart H, Weiner DE, Griffith J, Salem D, Levey AS, Sarnak MJ: **Waist-to-Hip Ratio and Body Mass Index as Risk Factors for Cardiovascular Events in CKD.** *Am J Kidney Dis* 2008, **52:**49-57.
183. Fahrleitner-Pammer A, Herberth J, Browning SR, Obermayer-Pietsch B, Wirnsberger G, Holzer H, Dobnig H, Malluche HH: **Bone markers predict cardiovascular events in chronic kidney disease.** *J Bone Miner Res* 2008, **23:**1850-1858.
184. Obermayr RP, Temml C, Gutjahr G, Kainz A, Klauser-Braun R, Függer R, Oberbauer R: **Body mass index modifies the risk of cardiovascular death in proteinuric chronic kidney disease.** *Nephrol Dial Transplant* 2009, **24:**2421-2428.

185. Shlipak MG, Fried LF, Cushman M, Manolio TA, Peterson D, Stehman-Breen C, Bleyer A, Newman A, Siscovick D, Psaty B: **Cardiovascular mortality risk in chronic kidney disease: Comparison of traditional and novel risk factors.** *JAMA* 2005, **293:**1737-1745.
186. Mehrotra R, Kermah DA, Salusky IB, Wolf MS, Thadhani RI, Chiu YW, Martins D, Adler SG, Norris KC: **Chronic kidney disease, hypovitaminosis D, and mortality in the United States.** *Kidney Int* 2009, **76:**977-983.
187. Anavekar NS, Gans DJ, Berl T, Rohde RD, Cooper W, Bhaumik A, Hunsicker LG, Rouleau JL, Lewis JB, Rosendorff C, et al: **Predictors of cardiovascular events in patients with type 2 diabetic nephropathy and hypertension: A case for albuminuria.** *Kidney Int Suppl* 2004, **66:**S50-S55.
188. Astor BC, Shafi T, Hoogeveen RC, Matsushita K, Ballantyne CM, Inker LA, Coresh J: **Novel markers of kidney function as predictors of ESRD, cardiovascular disease, and mortality in the general population.** *Am J Kidney Dis* 2012, **59:**653-662.
189. Baek SD, Baek CH, Kim JS, Kim SM, Kim JH, Kim SB: **Does stage III chronic kidney disease always progress to end-stage renal disease? A ten-year follow-up study.** *Scand J Urol Nephrol* 2012, **46:**232-238.
190. Busch M, Franke S, Wolf G, Brandstädt A, Ott U, Gerth J, Hunsicker LG, Stein G: **The Advanced Glycation End Product N ε-Carboxymethyllysine Is Not a Predictor of Cardiovascular Events and Renal Outcomes in Patients With Type 2 Diabetic Kidney Disease and Hypertension.** *Am J Kidney Dis* 2006, **48:**571-579.
191. Carrero JJ, Kyriazis J, Sonmez A, Tzanakis I, Qureshi AR, Stenvinkel P, Saglam M, Stylianou K, Yaman H, Taslipinar A, et al: **Prolactin levels, endothelial dysfunction, and the risk of cardiovascular events and mortality in patients with CKD.** *Clin J Am Soc Nephrol* 2012, **7:**207-215.
192. Chen FA, Yang CY, Yang WC, Chen JY, Ng YY, Li SY, Liu WS, Cheng ST, Wang YJ, Lin CC: **Ankle-brachial index is a powerful predictor of renal outcome and cardiovascular events in patients with chronic kidney disease.** *ScientificWorldJournal* 2012, **2012**.
193. Meijers BKI, Claes K, Bammens B, De Loor H, Viaene L, Verbeke K, Kuypers D, Vanrenterghem Y, Evenepoel P: **p-Cresol and cardiovascular risk in mild-to-moderate kidney disease.** *Clin J Am Soc Nephrol* 2010, **5:**1182-1189.
194. Peralta CA, Shlipak MG, Fan D, Ordoñez J, Lash JP, Chertow GM, Go AS: **Risks for end-stage renal disease, cardiovascular events, and death in hispanic versus non-hispanic white adults with chronic kidney disease.** *J Am Soc Nephrol* 2006, **17:**2892-2899.
195. Sakuma M, Nakamura M, Tanaka F, Onoda T, Itai K, Tanno K, Ohsawa M, Sakata K, Yoshida Y, Kawamura K, et al: **Plasma B-type natriuretic peptide level and cardiovascular events in chronic kidney disease in a community-based population.** *Circ J* 2010, **74:**792-797.
196. Sasso FC, Chiodini P, Carbonara O, De Nicola L, Conte G, Salvatore T, Nasti R, Marfella R, Gallo C, Signoriello S, et al: **High cardiovascular risk in patients with Type 2 diabetic nephropathy: The predictive role of albuminuria and glomerular filtration rate. The NID-2 Prospective Cohort Study.** *Nephrol Dial Transplant* 2012, **27:**2269-2274.
197. Shepherd J, Kastelein JJP, Bittner VA, Carmena R, Deedwania PC, Breazna A, Dobson S, Wilson DJ, Zuckerman AL, Wenger NK: **Intensive lipid lowering with atorvastatin in patients with coronary artery disease, diabetes, and chronic kidney disease.** *Mayo Clin Proc* 2008, **83:**870-879.
198. Weiner DE, Krassilnikova M, Tighiouart H, Salem DN, Levey AS, Sarnak MJ: **CKD classification based on estimated GFR over three years and subsequent cardiac and mortality outcomes: A cohort study.** *BMC Nephrol* 2009, **10**.
199. Zoungas S, Cameron JD, Kerr PG, Wolfe R, Muske C, McNeil JJ, McGrath BP: **Association of Carotid Intima-Medial Thickness and Indices of Arterial Stiffness With Cardiovascular Disease Outcomes in CKD.** *Am J Kidney Dis* 2007, **50:**622-630.
200. Alarcón GS, McGwin Jr G, Petri M, Ramsey-Goldman R, Fessler BJ, Vilá LM, Edberg JC, Reveille JD, Kimberly RP: **Time to renal disease and end-stage renal disease in PROFILE: A multiethnic lupus cohort.** *PLoS Med* 2006, **3:**1949-1956.
201. Chan JCN, Wat N, So WY, Lam KSL, Chua CT, Wong KS, Morad Z, Dickson TZ, Hille D, Zhang Z, et al: **Renin Angiotensin Aldosterone System Blockade and Renal Disease in Patients with Type 2 Diabetes: An Asian perspective from the RENAAL study.** *Diabetes Care* 2004, **27:**874-879.
202. Chandra P, Sands RL, Gillespie BW, Levin NW, Kotanko P, Kiser M, Finkelstein F, Hinderliter A, Pop-Busui R, Rajagopalan S, Saran R: **Predictors of heart rate variability and its prognostic significance in chronic kidney disease.** *Nephrol Dial Transplant* 2012, **27:**700-709.
203. Choi AI, Rodriguez RA, Bacchetti P, Bertenthal D, Volberding PA, O'Hare AM: **Racial differences in end-stage renal disease rates in HIV infection versus diabetes.** *J Am Soc Nephrol* 2007, **18:**2968-2974.
204. Conley J, Olafsson A, Djamali A: **Do statins delay the incidence of ESRD in diabetic patients with moderate CKD?** *J Nephrol* 2010, **23:**321-327.

205. Eijkelkamp WBA, Zhang Z, Remuzzi G, Parving HH, Cooper ME, Keane WF, Shahinfar S, Gleim GW, Weir MR, Brenner BM, De Zeeuw D: **Albuminuria is a target for renoprotective therapy independent from blood pressure in patients with type 2 diabetic nephropathy: Post hoc analysis from the reduction of endpoints in NIDDM with the angiotensin II antagonist losartan (RENAAL) trial.** *J Am Soc Nephrol* 2007, **18:**1540-1546.
206. Gansevoort RT, Matsushita K, Van Der Velde M, Astor BC, Woodward M, Levey AS, Jong PED, Coresh J: **Lower estimated GFR and higher albuminuria are associated with adverse kidney outcomes. A collaborative meta-analysis of general and high-risk population cohorts.** *Kidney Int* 2011, **80:**93-104.
207. Iseki K, Ikemiya Y, Inoue T, Iseki C, Kinjo K, Takishita S: **Significance of hyperuricemia as a risk factor for developing ESRD in a screened cohort.** *Am J Kidney Dis* 2004, **44:**642-650.
208. Iseki K, Tozawa M, Ikemiya Y, Kinjo K, Iseki C, Takishita S: **Relationship between dyslipidemia and the risk of developing end-stage renal disease in a screened cohort.** *Clin Exp Nephrol* 2005, **9:**46-52.
209. Jafar TH, Schmid CH, Stark PC, Toto R, Remuzzi G, Ruggenenti P, Marcantoni C, Becker G, Shahinfar S, de Jong PE, et al: **The rate of progression of renal disease may not be slower in women compared with men: A patient-level meta-analysis.** *Nephrol Dial Transplant* 2003, **18:**2047-2053.
210. Jotwani V, Li Y, Grunfeld C, Choi AI, Shlipak MG: **Risk factors for ESRD in HIV-infected individuals: Traditional and HIV-related factors.** *Am J Kidney Dis* 2012, **59:**628-635.
211. Kollerits B, Fliser D, Heid IM, Ritz E, Kronenberg F: **Gender-specific association of adiponectin as a predictor of progression of chronic kidney disease: The Mild to Moderate Kidney Disease Study.** *Kidney Int* 2007, **71:**1279-1286.
212. Komatsu H, Fujimoto S, Sato Y, Hara S, Yamada K, Morita S, Eto T: **"Point of no return (PNR)" in progressive IgA nephropathy: Significance of blood pressure and proteinuria management up to PNR.** *J Nephrol* 2005, **18:**690-695.
213. Kop WJ, Seliger SL, Fink JC, Katz R, Odden MC, Fried LF, Rifkin DE, Sarnak MJ, Gottdiener JS: **Longitudinal association of depressive symptoms with rapid kidney function decline and adverse clinical renal disease outcomes.** *Clin J Am Soc Nephrol* 2011, **6:**834-844.
214. Kuo HW, Tsai SS, Tiao MM, Liu YC, Lee IM, Yang CY: **Analgesic use and the risk for progression of chronic kidney disease.** *Pharmacoepidemiol Drug Saf* 2010, **19:**745-751.
215. Levey AS, Greene T, Sarnak MJ, Wang X, Beck GJ, Kusek JW, Collins AJ, Kopple JD: **Effect of Dietary Protein Restriction on the Progression of Kidney Disease: Long-Term Follow-Up of the Modification of Diet in Renal Disease (MDRD) Study.** *Am J Kidney Dis* 2006, **48:**879-888.
216. Liu M, Shi S, Senthilnathan S, Yu J, Wu E, Bergmann C, Zerres K, Bogdanova N, Coto E, Deltas C, et al: **Genetic variation of DKK3 may modify renal disease severity in ADPKD.** *J Am Soc Nephrol* 2010, **21:**1510-1520.
217. Mohanram A, Zhang Z, Shahinfar S, Keane WF, Brenner BM, Toto RD: **Anemia and end-stage renal disease in patients with type 2 diabetes and nephropathy.** *Kidney Int* 2004, **66:**1131-1138.
218. Nakayama M, Sato T, Miyazaki M, Matsushima M, Sato H, Taguma Y, Ito S: **Increased risk of cardiovascular events and mortality among non-diabetic chronic kidney disease patients with hypertensive nephropathy: The Gonryo study.** *Hypertens Res* 2011, **34:**1106-1110.
219. Omae K, Ogawa T, Nitta K: **Therapeutic advantage of angiotensin-converting enzyme inhibitors in patients with proteinuric chronic kidney disease.** *Heart Vessels* 2010, **25:**203-208.
220. Paoletti E, Bellino D, Gallina AM, Amidone M, Cassottana P, Cannella G: **Is left ventricular hypertrophy a powerful predictor of progression to dialysis in chronic kidney disease?** *Nephrol Dial Transplant* 2011, **26:**670-677.
221. Radican L, Wartenberg D, Rhoads GG, Schneider D, Wedeen R, Stewart P, Blair A: **A retrospective occupational cohort study of end-stage renal disease in aircraft workers exposed to trichloroethylene and other hydrocarbons.** *J Occup Environ Med* 2006, **48:**1-12.
222. Reed BY, McFann K, Reza Bekheirnia M, Nobkhthaghighi N, Masoumi A, Johnson AM, Abdollah Shamshiraz A, Kelleher CL, Schrier RW: **Variation in Age at ESRD in Autosomal Dominant Polycystic Kidney Disease.** *Am J Kidney Dis* 2008, **51:**173-183.
223. Roblin DW, Khoury A, Pisanelli W, Dahar W, Roth M: **Risk for Incident Renal Dialysis in a Managed Care Population.** *Am J Kidney Dis* 2006, **48:**205-211.

224. Ruggenenti P, Perticucci E, Cravedi P, Gambara V, Costantini M, Sharma SK, Perna A, Remuzzi G: **Role of remission clinics in the longitudinal treatment of CKD.** *J Am Soc Nephrol* 2008, **19:**1213-1224.
225. Sakaguchi Y, Shoji T, Hayashi T, Suzuki A, Shimizu M, Mitsumoto K, Kawabata H, Niihata K, Okada N, Isaka Y, et al: **Hypomagnesemia in type 2 diabetic nephropathy: A novel predictor of end-stage renal disease.** *Diabetes Care* 2012, **35:**1591-1597.
226. Sarnak MJ, Greene T, Wang X, Beck G, Kusek JW, Collins AJ, Levey AS: **The effect of a lower target blood pressure on the progression of kidney disease: Long-term follow-up of the modification of diet in renal disease study.** *Ann Intern Med* 2005, **142:**342-351.
227. Shahinfar S, Dickson TZ, Ahmed T, Zhang Z, Ramjit D, Smith RD, Brenner BM: **Losartan in patients with type 2 diabetes and proteinuria: Observations from the RENAAL Study.** *Kidney Int Suppl* 2002, **62:**S64-S67.
228. Tangri N, Stevens LA, Griffith J, Tighiouart H, Djurdjev O, Naimark D, Levin A, Levey AS: **A predictive model for progression of chronic kidney disease to kidney failure.** *JAMA* 2011, **305:**1553-1559.
229. Tazón-Vega B, Vilardell M, Pérez-Oller L, Ars E, Ruiz P, Devuyst O, Lens X, Fernández-Llama P, Ballarín J, Torra R: **Study of candidate genes affecting the progression of renal disease in autosomal dominant polycystic kidney disease type 1.** *Nephrol Dial Transplant* 2007, **22:**1567-1577.
230. Tsui JI, Vittinghoff E, Shlipak MG, Bertenthal D, Inadomi J, Rodriguez RA, O'Hare AM: **Association of hepatitis C seropositivity with increased risk for developing end-stage renal disease.** *Arch Intern Med* 2007, **167:**1271-1276.
231. Welander A, Prütz KG, Fored M, Ludvigsson JF: **Increased risk of end-stage renal disease in individuals with coeliac disease.** *Gut* 2012, **61:**64-68.
232. Wright Jr JT, Bakris G, Greene T, Agodoa LY, Appel LJ, Charleston J, Cheek D, Douglas-Baltimore JG, Gassman J, Glassock R, et al: **Effect of blood pressure lowering and antihypertensive drug class on progression of hypertensive kidney disease: Results from the AASK trial.** *JAMA* 2002, **288:**2421-2431.
233. Okonogi H, Utsunomiya Y, Miyazaki Y, Koike K, Hirano K, Tsuboi N, Suzuki T, Hara Y, Ogura M, Hosoya T, Kawamura T: **A predictive clinical grading system for immunoglobulin a nephropathy by combining proteinuria and estimated glomerular filtration rate.** *Nephron Clin Pract* 2011, **118:**c292-c300.
234. Banerjee D, Brincat S, Gregson H, Contreras G, Streather C, Oliveira D, Nelson S: **Pulse pressure and inhibition of renin-angiotensin system in chronic kidney disease.** *Nephrol Dial Transplant* 2006, **21:**975-978.
235. Berthoux FC, Berthoux P, Mariat C, Thibaudin L, Afiani A, Linossier MT: **CC-chemokine receptor five gene polymorphism in primary IgA nephropathy: The 32 bp deletion allele is associated with late progression to end-stage renal failure with dialysis.** *Kidney Int* 2006, **69:**565-572.
236. Evans M, Fored CM, Nise G, Bellocco R, Nyrén O, Elinder CG: **Occupational Lead Exposure and Severe CKD: A Population-Based Case-Control and Prospective Observational Cohort Study in Sweden.** *Am J Kidney Dis* 2010, **55:**497-506.
237. Jones C, Roderick P, Harris S, Rogerson M: **An evaluation of a shared primary and secondary care nephrology service for managing patients with moderate to advanced CKD.** *Am J Kidney Dis* 2006, **47:**103-114.
238. Magistroni R, He N, Wang K, Andrew R, Johnson A, Gabow P, Dicks E, Parfrey P, Torra R, San-Millan JL, et al: **Genotype-renal function correlation in type 2 autosomal dominant polycystic kidney disease.** *J Am Soc Nephrol* 2003, **14:**1164-1174.
239. Minutolo R, Chiodini P, Cianciaruso B, Pota A, Bellizzi V, Avino D, Mascia S, Laurino S, Bertino V, Conte G, De Nicola L: **Epoetin therapy and hemoglobin level variability in nondialysis patients with chronic kidney disease.** *Clin J Am Soc Nephrol* 2009, **4:**552-559.
240. Noureddine LA, Usman SA, Yu Z, Moorthi RN, Moe SM: **Hepatitis C increases the risk of progression of chronic kidney disease in patients with glomerulonephritis.** *Am J Nephrol* 2010, **32:**311-316.
241. Rabbani MA, Ali SS, Murtaza G, Ahmad B, Maria Q, Siddiqui BK, Shah SMA, Ahmad A: **Clinical presentation and outcome of Autosomal Dominant Polycystic Kidney Disease in Pakistan: A single center experience.** *J Pak Med Assoc* 2008, **58:**305-309.
242. Roshanravan B, Khatri M, Robinson-Cohen C, Levin G, Patel KV, de Boer IH, Seliger S, Ruzinski J, Himmelfarb J, Kestenbaum B: **A Prospective Study of Frailty in Nephrology-Referred Patients With CKD.** *Am J Kidney Dis* 2012.
243. Smith DH, Johnson ES, Thorp ML, Yang X, Neil N: **Hyperparathyroidism in chronic kidney disease: A retrospective cohort study of costs and outcomes.** *J Bone Miner Metab* 2009, **27:**287-294.

244. Tsai YC, Hung CC, Hwang SJ, Wang SL, Hsiao SM, Lin MY, Kung LF, Hsiao PN, Chen HC: **Quality of life predicts risks of end-stage renal disease and mortality in patients with chronic kidney disease.** *Nephrol Dial Transplant* 2010, **25:**1621-1626.
245. Atthobari J, Brantsma AH, Gansevoort RT, Visser ST, Asselbergs FW, van Gilst WH, de Jong PE, de Jong-van den Berg LTW: **The effect of statins on urinary albumin excretion and glomerular filtration rate: Results from both a randomized clinical trial and an observational cohort study.** *Nephrol Dial Transplant* 2006, **21:**3106-3114.
246. Cheng HT, Huang JW, Chiang CK, Yen CJ, Hung KY, Wu KD: **Metabolic syndrome and insulin resistance as risk factors for development of chronic kidney disease and rapid decline in renal function in elderly.** *J Clin Endocrinol Metab* 2012, **97:**1268-1276.
247. Coppo R, Peruzzi L, Amore A, Piccoli A, Cochat P, Stone R, Kirschstein M, Linné T: **IgACE: A placebo-controlled, randomized trial of angiotensin-converting enzyme inhibitors in children and young people with IgA nephropathy and moderate proteinuria.** *J Am Soc Nephrol* 2007, **18:**1880-1888.
248. Goicoechea M, De Vinuesa SG, Verdalles U, Ruiz-Caro C, Ampuero J, Rincón A, Arroyo D, Luño J: **Effect of allopurinol in chronic kidney disease progression and cardiovascular risk.** *Clin J Am Soc Nephrol* 2010, **5:**1388-1393.
249. Vupputuri S, Batuman V, Muntner P, Bazzano LA, Lefante JJ, Whelton PK, He J: **Effect of Blood Pressure on Early Decline in Kidney Function among Hypertensive Men.** *Hypertension* 2003, **42:**1144-1149.
250. Asrani SK, Buchanan P, Pinsky B, Rey LR, Schnitzler M, Kanwal F: **Lack of Association Between Hepatitis C Infection and Chronic Kidney Disease.** *Clin Gastroenterol Hepatol* 2010, **8:**79-84.
251. Bomback AS, Katz R, He K, Shoham DA, Burke GL, Klemmer PJ: **Sugar-sweetened beverage consumption and the progression of chronic kidney disease in the Multi-Ethnic Study of Atherosclerosis (MESA).** *Am J Clin Nutr* 2009, **90:**1172-1178.
252. Fried L, Solomon C, Shlipak M, Seliger S, Stehman-Breen C, Bleyer AJ, Chaves P, Furberg C, Kuller L, Newman A: **Inflammatory and prothrombotic markers and the progression of renal disease in elderly individuals.** *J Am Soc Nephrol* 2004, **15:**3184-3191.
253. Fried LF, Katz R, Cushman M, Sarnak M, Shlipak MG, Kuller L, Newman AB: **Change in cardiovascular risk factors with progression of kidney disease.** *Am J Nephrol* 2009, **29:**334-341.
254. Idasiak-Piechocka I, Oko A, Pawliczak E, Kaczmarek E, Czekalsk S: **Urinary excretion of soluble tumour necrosis factor receptor 1 as a marker of increased risk of progressive kidney function deterioration in patients with primary chronic glomerulonephritis.** *Nephrol Dial Transplant* 2010, **25:**3948-3956.
255. Knight EL, Stampfer MJ, Hankinson SE, Spiegelman D, Curhan GC: **The Impact of Protein Intake on Renal Function Decline in Women with Normal Renal Function or Mild Renal Insufficiency.** *Ann Intern Med* 2003, **138:**460-467+I451.
256. Orth SR, Schroeder T, Ritz E, Ferrari P: **Effects of smoking on renal function in patients with type 1 and type 2 diabetes mellitus.** *Nephrol Dial Transplant* 2005, **20:**2414-2419.
257. White SL, Polkinghorne KR, Cass A, Shaw JE, Atkins RC, Chadban SJ: **Alcohol consumption and 5-year onset of chronic kidney disease: The AusDiab study.** *Nephrol Dial Transplant* 2009, **24:**2464-2472.
258. Hanai K, Babazono T, Yoshida N, Nyumura I, Toya K, Hayashi T, Bouchi R, Tanaka N, Ishii A, Iwamoto Y: **Gender differences in the association between HDL cholesterol and the progression of diabetic kidney disease in type 2 diabetic patients.** *Nephrol Dial Transplant* 2012, **27:**1070-1075.
259. LaMattina JC, Foley DP, Mezrich JD, Fernandez LA, Vidyasagar V, D'Alessandro AM, Musat AI, Samaniego-Picota MD, Pascual J, Alejandro MDR, et al: **Chronic kidney disease stage progression in liver transplant recipients.** *Clin J Am Soc Nephrol* 2011, **6:**1851-1857.
260. Palmas W, Pickering T, Eimicke JP, Moran A, Teresi J, Schwartz JE, Field L, Weinstock RS, Shea S: **Value of Ambulatory Arterial Stiffness Index and 24-h Pulse Pressure to Predict Progression of Albuminuria in Elderly People With Diabetes Mellitus.** *Am J Hypertens* 2007, **20:**493-500.
261. Van Pottelbergh G, Bartholomeeusen S, Buntinx F, Degryse J: **The evolution of renal function and the incidence of end-stage renal disease in patients aged ≥50 years.** *Nephrol Dial Transplant* 2012, **27:**2297-2303.
262. Cummings DM, Larsen LC, Doherty L, Lea CS, Holbert D: **Glycemic control patterns and kidney disease progression among primary care patients with diabetes mellitus.** *J Am Board Fam Med* 2011, **24:**391-398.

263. Iwasaki M, Taylor GW, Nesse W, Vissink A, Yoshihara A, Miyazaki H: **Periodontal disease and decreased kidney function in Japanese elderly.** *Am J Kidney Dis* 2012, **59:**202-209.
264. Meguro S, Shigihara T, Kabeya Y, Tomita M, Atsumi Y: **Increased risk of renal deterioration associated with low e-GFR in type 2 diabetes mellitus only in albuminuric subjects.** *Intern Med* 2009, **48:**657-663.
265. Nishiyama K, Tanaka Y, Nakajima K, Mokubo A, Atsumi Y, Matsuoka K, Watada H, Hirose T, Nomiyama T, Maeda S, Kawamori R: **Polymorphism of the solute carrier family 12 (sodium/chloride transporters) member 3, SLC12A3, gene at exon 23 (+78G/A: Arg913Gln) is associated with elevation of urinary albumin excretion in Japanese patients with type 2 diabetes: A 10-year longitudinal study.** *Diabetologia* 2005, **48:**1335-1338.
266. Descamps-Latscha B, Witko-Sarsat V, Nguyen-Khoa T, Nguyen AT, Gausson V, Mothu N, Cardoso C, Noël LH, Guérin AP, London GM, Jungers P: **Early prediction of IgA nephropathy progression: Proteinuria and AOPP are strong prognostic markers.** *Kidney Int* 2004, **66:**1606-1612.
267. Okada T, Matsumoto H, Nagaoka Y, Nakao T: **Association of home blood pressure variability with progression of chronic kidney disease.** *Blood Press Monit* 2012, **17:**1-7.
268. Szczech LA, Gange SJ, Van der Horst C, Bartlett JA, Young M, Cohen MH, Anastos K, Klassen PS, Svetkey LP: **Predictors of proteinuria and renal failure among women with HIV infection.** *Kidney Int* 2002, **61:**195-202.
269. Yamada H, Suga N, Maeda K, Kimura Y, Miura N, Futenma A, Imai H: **Effects of combination therapy with angiotensin II type I receptor blockers and calcium channel blockers on renal function in hypertensive patients: A retrospective, "real-world" comparative study.** *Arzneimittelforschung* 2010, **60:**64-70.
270. Song KH, Ko SH, Kim HW, Ahn YB, Lee JM, Son HS, Yoon KH, Cha BY, Lee KW, Son HY: **Prospective study of lipoprotein(a) as a risk factor for deteriorating renal function in type 2 diabetic patients with overt proteinuria.** *Diabetes Care* 2005, **28:**1718-1723.
271. Agarwal R, Light RP: **Patterns and prognostic value of total and differential leukocyte count in chronic kidney disease.** *Clin J Am Soc Nephrol* 2011, **6:**1393-1399.
272. Boes E, Fliser D, Ritz E, König P, Lhotta K, Mann JFE, Müller GA, Neyer U, Riegel W, Riegler P, Kronenberg F: **Apolipoprotein A-IV predicts progression of chronic kidney disease: The mild to moderate kidney disease study.** *J Am Soc Nephrol* 2006, **17:**528-536.
273. Chen Y, Salem RM, Rao F, Fung MM, Bhatnagar V, Pandey B, Mahata M, Waalen J, Nievergelt CM, Lipkowitz MS, et al: **Common charge-shift mutation Glu65Lys in K + channel β 1-subunit KCNMB1: Pleiotropic consequences for glomerular filtration rate and progressive renal disease.** *Am J Nephrol* 2010, **32:**414-424.
274. Dieplinger B, Mueller T, Kollerits B, Struck J, Ritz E, Von Eckardstein A, Haltmayer M, Kronenberg F: **Pro-A-type natriuretic peptide and pro-adrenomedullin predict progression of chronic kidney disease: The MMKD Study.** *Kidney Int* 2009, **75:**408-414.
275. Fliser D, Kollerits B, Neyer U, Ankerst DP, Lhotta K, Lingenhel A, Ritz E, Kronenberg F: **Fibroblast Growth Factor 23 (FGF23) predicts progression of chronic kidney disease: The Mild to Moderate Kidney Disease (MMKD) study.** *J Am Soc Nephrol* 2007, **18:**2601-2608.
276. Goyal A, Crook ED: **Thiazolidinediones and progression of renal disease in patients with diabetes.** *J Investig Med* 2006, **54:**56-61.
277. Hunsicker LG, Atkins RC, Lewis JB, Braden G, Champion De Crespigny PJ, DeFerrari G, Drury P, Locatelli F, Wiegmann TB, Lewis EJ: **Impact of irbesartan, blood pressure control, and proteinuria on renal outcomes in the Irbesartan Diabetic Nephropathy Trial.** *Kidney Int Suppl* 2004, **66:**S99-S101.
278. Hwang HS, Kim BS, Shin YS, Yoon HE, Song JC, Choi BS, Park CW, Yang CW, Kim YS, Bang BK: **Predictors for progression in immunoglobulin A nephropathy with significant proteinuria.** *Nephrology (Carlton)* 2010, **15:**236-241.
279. Jacob S, Héry M, Protois JC, Rossert J, Stengel B: **Effect of organic solvent exposure on chronic kidney disease progression: The GN-PROGRESS cohort study.** *J Am Soc Nephrol* 2007, **18:**274-281.
280. Jafar TH, Stark PC, Schmid CH, Landa M, Maschio G, De Jong PE, De Zeeuw D, Shahinfar S, Toto R, Levey AS: **Progression of Chronic Kidney Disease: The Role of Blood Pressure Control, Proteinuria, and Angiotensin-Converting Enzyme Inhibition. A Patient-Level Meta-Analysis.** *Ann Intern Med* 2003, **139:**244-252+I236.
281. Jafar TH, Stark PC, Schmid CH, Strandgaard S, Kamper AL, Maschio G, Becicer G, Perrone RD, Levey AS: **The effect of angiotensin-converting-enzyme inhibitors on progression of advanced polycystic kidney disease.** *Kidney Int* 2005, **67:**265-271.

282. Moon SJ, Bae KS, Park HC, Kim JK, Park JT, Lee JE, Rim SJ, Ha SK: **The effect of anemia and left ventricular geometric patterns on renal disease progression in type 2 diabetic nephropathy.** *J Nephrol* 2011, **24:**50-59.
283. Nakao N, Seno H, Kasuga H, Toriyama T, Kawahara H, Fukagawa M: **Effects of combination treatment with losartan and trandolapril on office and ambulatory blood pressures in non-diabetic renal disease: A COOPERATE-ABP substudy (Retraction in: American Journal of Nephrology (2009) 30:6 (563)).** *Am J Nephrol* 2004, **24:**543-548.
284. Okada T, Nagao T, Matsumoto H, Nagaoka Y, Wada T, Nakao T: **Histological predictors for renal prognosis in diabetic nephropathy in diabetes mellitus type 2 patients with overt proteinuria.** *Nephrology (Carlton)* 2012, **17:**68-75.
285. Peters HPE, Waanders F, Meijer E, Van Den Brand J, Steenbergen EJ, Van Goor H, Wetzels JFM: **High urinary excretion of kidney injury molecule-1 is an independent predictor of end-stage renal disease in patients with IgA nephropathy.** *Nephrol Dial Transplant* 2011, **26:**3581-3588.
286. Schwarz S, Trivedi BK, Kalantar-Zadeh K, Kovesdy CP: **Association of disorders in mineral metabolism with progression of chronic kidney disease.** *Clin J Am Soc Nephrol* 2006, **1:**825-831.
287. Soares CM, Diniz JSS, Lima EM, Oliveira GR, Canhestro MR, Colosimo EA, E Silva ACS, Oliveira EA: **Predictive factors of progression to chronic kidney disease stage 5 in a predialysis interdisciplinary programme.** *Nephrol Dial Transplant* 2009, **24:**848-855.
288. Staples AO, Greenbaum LA, Smith JM, Gipson DS, Filler G, Warady BA, Martz K, Wong CS: **Association between clinical risk factors and progression of chronic kidney disease in children.** *Clin J Am Soc Nephrol* 2010, **5:**2172-2179.
289. Sturm G, Kollerits B, Neyer U, Ritz E, Kronenberg F: **Uric acid as a risk factor for progression of non-diabetic chronic kidney disease? The Mild to Moderate Kidney Disease (MMKD) Study.** *Exp Gerontol* 2008, **43:**347-352.
290. Yang XL, So WY, Kong APS, Clarke P, Ho CS, Lam CWK, Ng MHL, Lyu RR, Yin DD, Chow CC, et al: **End-stage renal disease risk equations for Hong Kong Chinese patients with type 2 diabetes: Hong Kong Diabetes Registry.** *Diabetologia* 2006, **49:**2299-2308.
291. Yu CC, Lin JL, Lin-Tan DT: **Environmental Exposure to Lead and Progression of Chronic Renal Diseases: A Four-Year Prospective Longitudinal Study.** *J Am Soc Nephrol* 2004, **15:**1016-1022.
292. Scialla JJ, Appel LJ, Astor BC, Miller Iii ER, Beddhu S, Woodward M, Parekh RS, Anderson CAM: **Net endogenous acid production is associated with a faster decline in GFR in African Americans.** *Kidney Int* 2012, **82:**106-112.
293. Kim SB, Kwon S, Lee SK, Park JS: **Association of Atherosclerotic Coronary Artery Disease and Pulse Pressure with Renal Disease Progression.** *Ren Fail* 2003, **25:**1019-1027.
294. Caplin B, Nitsch D, Gill H, Hoefield R, Blackwell S, MacKenzie D, Cooper JA, Middleton RJ, Talmud PJ, Veitch P, et al: **Circulating methylarginine levels and the decline in renal function in patients with chronic kidney disease are modulated by DDAH1 polymorphisms.** *Kidney Int* 2010, **77:**459-467.
295. Caron M, Hudson M, Baron M, Nessim S, Steele R, Pope J, Markland J, Robinson D, Jones N, Khalidi N, et al: **Longitudinal study of renal function in systemic sclerosis.** *J Rheumatol* 2012, **39:**1829-1834.
296. Elung-Jensen T, Strandgaard S, Kamper AL: **Longitudinal observations on circadian blood pressure variation in chronic kidney disease stages 3-5.** *Nephrol Dial Transplant* 2008, **23:**2873-2878.
297. Fung MM, Chen Y, Lipkowitz MS, Salem RM, Bhatnagar V, Mahata M, Nievergelt CM, Rao F, Mahata SK, Schork NJ, et al: **Adrenergic beta-1 receptor genetic variation predicts longitudinal rate of GFR decline in hypertensive nephrosclerosis.** *Nephrol Dial Transplant* 2009, **24:**3677-3686.
298. Gupta SK, Smurzynski M, Franceschini N, Bosch RJ, Szczech LA, Kalayjian RC: **The effects of HIV type-1 viral suppression and non-viral factors on quantitative proteinuria in the highly active antiretroviral therapy era.** *Antivir Ther* 2009, **14:**543-549.
299. Higashihara E, Torres VE, Chapman AB, Grantham JJ, Bae K, Watnick TJ, Horie S, Nutahara K, Ouyang J, Krasa HB, Czerwiec FS: **Tolvaptan in autosomal dominant polycystic kidney disease: Three years' experience.** *Clin J Am Soc Nephrol* 2011, **6:**2499-2507.
300. Kalayjian RC, Franceschini N, Gupta SK, Szczech LA, Mupere E, Bosch RJ, Smurzynski M, Albert JM: **Suppression of HIV-1 replication by antiretroviral therapy improves renal function in persons with low CD4 cell counts and chronic kidney disease.** *AIDS* 2008, **22:**481-487.
301. Kobayashi M, Hirawa N, Morita S, Yatsu K, Kobayashi Y, Yamamoto Y, Saka S, Toya Y, Yasuda G, Umemura S: **Silent brain infarction and rapid decline of kidney function in patients with CKD: A prospective cohort study.** *Am J Kidney Dis* 2010, **56:**468-476.

302. Mahajan A, Simoni J, Sheather SJ, Broglio KR, Rajab MH, Wesson DE: **Daily oral sodium bicarbonate preserves glomerular filtration rate by slowing its decline in early hypertensive nephropathy.** *Kidney Int* 2010, **78:**303-309.
303. Mauss S, Berger F, Filmann N, Hueppe D, Henke J, Hegener P, Athmann C, Schmutz G, Herrmann E: **Effect of HBV polymerase inhibitors on renal function in patients with chronic hepatitis B.** *J Hepatol* 2011, **55:**1235-1240.
304. Rasch MG, Engsig FN, Feldt-Rasmussen B, Kirk O, Kronborg G, Pedersen C, Gerstoft J, Obel N: **Renal function and incidence of chronic kidney disease in HIV patients: A Danish cohort study.** *Scand J Infect Dis* 2012, **44:**689-696.
305. Von Känel R, Mausbach BT, Dimsdale JE, Mills PJ, Patterson TL, Ancoli-Israel S, Ziegler MG, Roepke SK, Chattillion EA, Allison M, Grant I: **Effect of chronic dementia caregiving and major transitions in the caregiving situation on kidney function: A longitudinal study.** *Psychosom Med* 2012, **74:**214-220.
306. Wang X, Lewis J, Appel L, Cheek D, Contreras G, Faulkner M, Feldman H, Gassman J, Lea J, Kopple J, et al: **Validation of creatinine-based estimates of GFR when evaluating risk factors in longitudinal studies of kidney disease.** *J Am Soc Nephrol* 2006, **17:**2900-2909.
307. Zhang P, Song PXK, Qu A, Greene T: **Efficient estimation for patient-specific rates of disease progression using nonnormal linear mixed models.** *Biometrics* 2008, **64:**29-38.
308. Liu M, Ying Z: **Joint analysis of longitudinal data with informative right censoring.** *Biometrics* 2007, **63:**363-371+633.
309. Xu L, Shao J: **Estimation in longitudinal or panel data models with random-effect-based missing responses.** *Biometrics* 2009, **65:**1175-1183.
310. Nanayakkara PWB, Van Guldener C, Ter Wee PM, Scheffer PG, Van Ittersum FJ, Twisk JW, Teerlink T, Van Dorp W, Stehouwer CDA: **Effect of a treatment strategy consisting of pravastatin, vitamin E, and homocysteine lowering on carotid intima-media thickness, endothelial function, and renal function in patients with mild to moderate chronic kidney disease: Results from the anti-oxidant therapy in chronic renal insufficiency (ATIC) study.** *Arch Intern Med* 2007, **167:**1262-1270.
311. Liu M, Lu W: **A semiparametric marginalized model for longitudinal data with informative dropout.** *J Probab Stat* 2012.
312. Schrier R, McFann K, Johnson A, Chapman A, Edelstein C, Brosnahan G, Ecder T, Tison L: **Cardiac and renal effects of standard versus rigorous blood pressure control in autosomal-dominant polycystic kidney disease: Results of a seven-year prospective randomized study.** *J Am Soc Nephrol* 2002, **13:**1733-1739.
313. Szeto CC, Chow KM, Kwan BCH, Chung KY, Leung CB, Li PKT: **Oral Calcitriol for the Treatment of Persistent Proteinuria in Immunoglobulin A Nephropathy: An Uncontrolled Trial.** *Am J Kidney Dis* 2008, **51:**724-731.
314. Tsui J, Vittinghoff E, Anastos K, Augenbraun M, Young M, Nowicki M, Cohen MH, Peters MG, Golub ET, Szczech L: **Hepatitis C Seropositivity and Kidney Function Decline Among Women With HIV: Data From the Women's Interagency HIV Study.** *Am J Kidney Dis* 2009, **54:**43-50.
315. Camilla R, Brachemi S, Pichette V, Cartier P, Laforest-Renald A, MacRae T, Madore F, Troyanov S: **Urinary monocyte chemotactic protein 1: Marker of renal function decline in diabetic and nondiabetic proteinuric renal disease.** *J Nephrol* 2011, **24:**60-67.
316. De Boer IH, Katz R, Fried LF, Ix JH, Luchsinger J, Sarnak MJ, Shlipak MG, Siscovick DS, Kestenbaum B: **Obesity and Change in Estimated GFR Among Older Adults.** *Am J Kidney Dis* 2009, **54:**1043-1051.
317. Fick-Brosnahan GM, Belz MM, McFann KK, Johnson AM, Schrier RW: **Relationship between renal volume growth and renal function in autosomal dominant polycystic kidney disease: A longitudinal study.** *Am J Kidney Dis* 2002, **39:**1127-1134.
318. Keller C, Katz R, Sarnak MJ, Fried LF, Kestenbaum B, Cushman M, Shlipak MG: **Inflammatory biomarkers and decline in kidney function in the elderly: The Cardiovascular Health Study.** *Nephrol Dial Transplant* 2010, **25:**119-124.
319. Khedr A, Khedr E, House AA: **Body Mass Index and the Risk of Progression of Chronic Kidney Disease.** *J Ren Nutr* 2011, **21:**455-461.
320. Lane C, Ashcroft A, Bothamley G, Yaqoob MM, Fan SLS: **Accelerated decline of GFR in diabetic nephropathy predicted by interferon release assay to tuberculosis antigens.** *Nephron Clin Pract* 2011, **117:**c266-c269.
321. Longenecker CT, Scherzer R, Bacchetti P, Lewis CE, Grunfeld C, Shlipak MG: **HIV viremia and changes in kidney function.** *AIDS* 2009, **23:**1089-1096.
322. Menon V, Katz R, Mukamal K, Kestenbaum B, De Boer IH, Siscovick DS, Sarnak MJ, Shlipak MG: **Alcohol consumption and kidney function decline in the elderly.** *Nephrol Dial Transplant* 2010, **25:**3301-3307.

323. Othman M, Kawar B, El Nahas AM: **Influence of obesity on progression of non-diabetic chronic kidney disease: A retrospective cohort study.** *Nephron Clin Pract* 2009, **113:**c16-c23.
324. Suzuki H, Nakamoto H, Okada H, Sugahara S, Kanno Y: **Self-measured systolic blood pressure in the morning is a strong indicator of decline of renal function in hypertensive patients with non-diabetic chronic renal insufficiency.** *Clin Exp Hypertens* 2002, **24:**249-260.
325. Tokiwa S, Muto S, China T, Horie S: **The relationship between renal volume and renal function in autosomal dominant polycystic kidney disease.** *Clin Exp Nephrol* 2011, **15:**539-545.
326. Tonelli M, Collins D, Robins S, Bloomfield H, Curhan GC: **Effect of gemfibrozil on change in renal function in men with moderate chronic renal insufficiency and coronary disease.** *Am J Kidney Dis* 2004, **44:**832-839.
327. Tonelli M, Moyé L, Sacks FM, Cole T, Curhan GC: **Effect of pravastatin on loss of renal function in people with moderate chronic renal insufficiency and cardiovascular disease.** *J Am Soc Nephrol* 2003, **14:**1605-1613.
328. Tonelli M, Sacks F, Pfeffer M, Jhangri GS, Curhan G: **Biomarkers of inflammation and progression of chronic kidney disease.** *Kidney Int* 2005, **68:**237-245.
329. Yokoyama H, Kanno S, Takahashi S, Yamada D, Honjo J, Saito K, Sone H, Haneda M: **Risks for glomerular filtration rate decline in association with progression of albuminuria in type 2 diabetes.** *Nephrol Dial Transplant* 2011, **26:**2924-2930.
330. Yokoyama H, Kanno S, Takahashi S, Yamada D, Itoh H, Saito K, Sone H, Haneda M: **Determinants of decline in glomerular filtration rate in nonproteinuric subjects with or without diabetes and hypertension.** *Clin J Am Soc Nephrol* 2009, **4:**1432-1440.
331. Yoshida T, Takei T, Shirota S, Tsukada M, Sugiura H, Itabashi M, Ogawa T, Uchida K, Tsuchiya K, Nitta K: **Risk factors for progression in patients with early-stage chronic kidney disease in the Japanese population.** *Intern Med* 2008, **47:**1859-1864.
332. Gooch K, Culleton BF, Manns BJ, Zhang J, Alfonso H, Tonelli M, Frank C, Klarenbach S, Hemmelgarn BR: **NSAID Use and Progression of Chronic Kidney Disease.** *Am J Med* 2007, **120:**280.e281-280.e287.
333. Lee YT, Chiu HC, Huang CT, Su HM, Wang CL, Lin TH, Voon WC, Chen HC, Lai WT, Sheu SH: **The A1166C polymorphism of angiotensin II type 1 receptor as a predictor of renal function decline over 4 years follow-up in an apparently healthy Chinese population.** *Clin Nephrol* 2009, **72:**457-467.
334. Lee YT, Chiu HC, Su HM, Voon WC, Lin TH, Lai WT, Sheu SH: **The D allele of angiotensin-converting enzyme polymorphism as a predictor of renal function decline over 4 years in an apparently healthy Chinese population aged 60 and older.** *J Am Geriatr Soc* 2008, **56:**718-723.
335. Arulkumaran N, Diwakar R, Tahir Z, Mohamed M, Kaski JC, Banerjee D: **Pulse pressure and progression of chronic kidney disease.** *J Nephrol* 2010, **23:**189-193.
336. Bastian HM, Alarcón GS, Roseman JM, McGwin Jr G, Vilá LM, Fessler BJ, Reveille JD, Ferńndez M, Andrade R, Sanchez ML, et al: **Systemic lupus erythematosus in a multiethnic US cohort (LUMINA) XL II: Factors predictive of new or worsening proteinuria.** *Rheumatology (Oxford)* 2007, **46:**683-689.
337. Bolignano D, Lacquaniti A, Coppolino G, Donato V, Campo S, Fazio MR, Nicocia G, Buemi M: **Neutrophil gelatinase-associated lipocalin (NGAL) and progression of chronic kidney disease.** *Clin J Am Soc Nephrol* 2009, **4:**337-344.
338. Bruno G, Biggeri A, Merletti F, Bargero G, Ferrero S, Pagano G, Perin PC: **Low incidence of end-stage renal disease and chronic renal failure in type 2 diabetes: A 10-year prospective study.** *Diabetes Care* 2003, **26:**2353-2358.
339. Buck J, Baker R, Cannaby AM, Nicholson S, Peters J, Warwick G: **Why do patients known to renal services still undergo urgent dialysis initiation? A cross-sectional survey.** *Nephrol Dial Transplant* 2007, **22:**3240-3245.
340. Daratha KB, Short RA, Corbett CF, Ring ME, Alicic R, Choka R, Tuttle KR: **Risks of subsequent hospitalization and death in patients with kidney disease.** *Clin J Am Soc Nephrol* 2012, **7:**409-416.
341. Dember LM, Hawkins PN, Hazenberg BPC, Gorevic PD, Merlini G, Butrimiene I, Livneh A, Lesnyak O, Puéchal X, Lachmann HJ, et al: **Eprodisate for the treatment of renal disease in AA amyloidosis.** *N Engl J Med* 2007, **356:**2349-2360.
342. Komatsu H, Fujimoto S, Kikuchi M, Sato Y, Kitamura K: **Tonsillectomy delays progression of advanced IgA nephropathy to end-stage kidney disease.** *Ren Fail* 2012, **34:**448-453.

343. Lee CC, Sun CY, Wu IW, Wang SY, Wu MS: **Metabolic syndrome loses its predictive power in late-stage chronic kidney disease progression - A paradoxical phenomenon.** *Clin Nephrol* 2011, **75:**141-149.
344. Lo LJ, Go AS, Chertow GM, McCulloch CE, Fan D, Ordõez JD, Hsu CY: **Dialysis-requiring acute renal failure increases the risk of progressive chronic kidney disease.** *Kidney Int* 2009, **76:**893-899.
345. Morita S, Fukuhara S, Akizawa T, Asano Y, Koshikawa S, Koide K, Kurokawa K: **Prognostic factors for a composite end-point of renal outcomes in patients with chronic kidney disease.** *Ther Apher Dial* 2006, **10:**72-77.
346. Panizo N, Goicoechea M, de Vinuesa SG, Arroyo D, Yuste C, Rincón A, Verdalles U, Ruiz-Caro C, Quiroga B, Luño J: **Chronic kidney disease progression in patients with autosomal dominant polycystic kidney disease.** *Nefrologia* 2012, **32:**197-205.
347. Sezer S, Karakan S, Acar NO: **Association of conicity index and renal progression in pre-dialysis chronic kidney disease.** *Ren Fail* 2012, **34:**165-170.
348. Sugiura T, Wada A: **Resistive index predicts renal prognosis in chronic kidney disease.** *Nephrol Dial Transplant* 2009, **24:**2780-2785.
349. Taskapan H, Tam P, Au V, Chow S, Fung J, Nagai G, Roscoe J, Ng P, Sikaneta T, Ting R, Oreopoulos DG: **Improvement in eGFR in patients with chronic kidney disease attending a nephrology clinic.** *Int Urol Nephrol* 2008, **40:**841-848.
350. Zhang AH, Tam P, Leblanc D, Zhong H, Chan CT, Bargman JM, Oreopoulos DG: **Natural history of CKD stage 4 and 5 patients following referral to renal management clinic.** *Int Urol Nephrol* 2009, **41:**977-982.
